# Prevention of Obesity Related Diseases through Laminarin-induced targeted delivery of Bindarit

**DOI:** 10.7150/thno.45788

**Published:** 2020-07-25

**Authors:** Chunmei Xu, Luqi Yin, Zhipeng Teng, Xuemei Zhou, Wenjie Li, Qiong Lai, Cuiping Peng, Chengyuan Zhang, Jie Lou, Xing Zhou

**Affiliations:** 1 School of Pharmacy and Bioengineering, Chongqing University of Technology, Chongqing 400054, China.; 2 Department of Pharmaceutics, College of Pharmacy, Third Military Medical University, Chongqing 400038, China.; 3 Department of Neurosurgery, Chongqing Traditional Chinese Medicine Hospital, Chongqing 400021, China.

**Keywords:** Nanomedicines, Oral Targeted Therapeutics, Obesity, Fatty Liver, Atherosclerosis

## Abstract

**Rationale:** The developement of oral targeted therapeutics for obesity and obesity-related diseases is challenging, as these diseases involve multiple lesions distributed throughout the whole body. Herein, we report a successful stragety for targeted oral delivery of bindarit to multiple obesity-related lesions including inflamed adipose tissue, fatty liver and atherosclerotic plaques.

**Methods:** The computer simulation from atomstic to mesoscale was first applied for designing bindarit-loaded nanoparticles (pBIN) and laminarin-modified bindarit-loaded nanoparticles (LApBIN). Then pBIN were suceesfully prepared using a dialysis procedure, and LApBIN were prepared though the interaction bewtween laminarin and pBIN. The physiochemical properties, *in vitro* and *in vivo* pharmacokinetics, oral targeting capability and in vivo efficacy of LApBIN in various obesity-related diseases were examined.

**Results:** LApBIN were sucessfully designed and prepared. Following oral administration of LApBIN, the nanoparticles could be sucessully orally adsorbed and translocated to monocytes. Contributed by the recruitment of monocytes to multiple obesity-related lesions, LApBIN successfully delivered bindarit to these lesions, and effectively suppressed inflammation there, which exerted successful preventive effects on high-fat-diet-induced obesity, insulin resistance, fatty liver and atherosclerosis.

**Conclusions:**This strategy could represent a promising approach to develop effective oral treatments for obesity and other metabolic diseases.

## Introduction

Obesity is a global public health issue, which results in many health complications. Furthermore, the condition is associated with comorbidities including type 2 diabetes mellitus, cardiovascular disease and fatty liver 1-4. Current anti-obesity therapeutics mostly focus on reducing food intake by suppressing the appetite 5. However, due to these therapeutics' serious mental and/or cardiovascular side effects, and limited efficacy for obesity-related diseases (ORDs), there is an urgent need for alternative treatment strategies 6. One of the most important discoveries to be made in this field over the last decade is the involvement of chronic inflammation in the pathogenesis of obesity and ORDs 7. It is well known that obesity, especially visceral fat accumulation, can lead to chronic low-grade inflammation. This promotes the initiation and progression of ORDs by stimulating the secretion of large amounts of pro-inflammatory cytokines including tumor necrosis factor-α (TNF-α), interleukin 1-β (IL1-β) and monocyte chemoattractant protein-1 (MCP-1) 8, 9; these pathological changes are interrelated, and are thus aggravated by each other 10-13.

Bindarit (BIN) is a selective inhibitor of MCP-1/CCL2 and TNF-α synthesis 14, 15, and has been proven to have an active role in reducing levels of triglyceride, cholesterol and glucose 16. This drug also has excellent therapeutic effects for tumors, coronary stent restenosis, acute pancreatitis, rheumatoid arthritis and other MCP-1/CCL2-induced inflammatory diseases 17-20. However, the low solubility, poor oral bioavailability and nonspecific distribution *in vivo* of BIN mean that high doses (around 200 mg/kg) are required for successful treatment of inflammation 21 and the risks of long-term treatment of obesity and ORDs with BIN are increased. If targeted delivered to the lesions to be achieved in obesity and ORDs, BIN-derived therapeutics could be used at low doses to treat obesity and ORDs.

Numerous targeted drug delivery systems have been developed and successfully used for the treatment of tumors, inflammation and atherosclerosis 22-24. Most targeted strategies are based on specific pathological characteristics of local lesions, such as specific enzymes, protein receptors or microenvironments 25, 26. However, in contrast to diseases with definite local lesions, there is no clear single lesion associated with obesity or ORDs, as these syndromes are a combination of over-weight status, atherosclerosis, insulin resistance, fatty liver and other symptoms. Although it is difficult to achieve the multiple-focus targeting that would be required to treat obesity and ORDs using traditional targeted drug delivery strategies, recent discovery of a link between the multiple lesions in obesity and ORDs has opened up avenues for targeted drug delivery. A recent study demonstrates that monocytes in the peripheral blood are recruited to inflammatory adipose tissue, fatty liver and atherosclerosis plaques 27; thus, these cells offer an approach for targeted delivery of BIN to each lesion in obesity and ORDs.

We have previously developed a bioinspired oral targeted delivery system using a yeast-based strategy for the treatment of tumors, atherosclerosis and rheumatoid arthritis based on the recruitment of monocytes in inflammation and tumors 28, 29. The present study aimed to develop an oral targeted delivery system based on a natural water-soluble β-1,3 glucan, laminarin (LAM), to deliver BIN to various lesions in obesity and ORDs via monocyte-mediated transportation. We demonstrate the successful preventive effects of this system for high-fat-diet-induced obesity, insulin resistance, fatty liver and atherosclerosis through delivery of a low dose of BIN (100 mg/kg once every 3 days).

## Results and Discussion

### Computational design of bindarit-loaded nanoparticles

**Figure 1:**
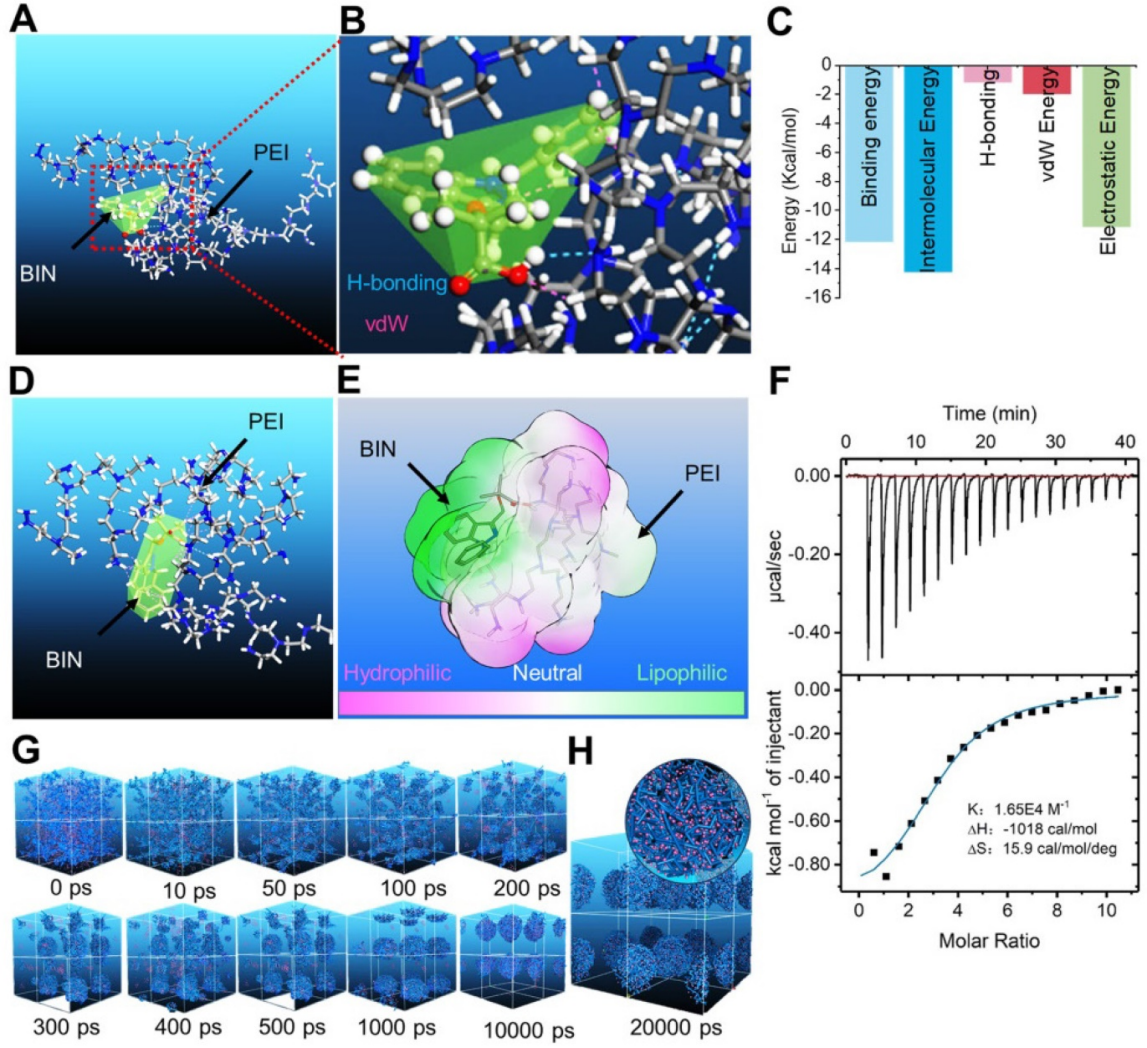
** Computational simulation to design BIN loaded nanoparticles.** (**A-B**) The lowest energy 3D conformation of BIN/PEI complex given by adsorption calculation. (**C**) The 2D conformation of BIN/PEI complex given by dock result calculated by Autodock 4.2. (**D**) The 3D conformation of BIN/PEI complex after 200 ns MD simulation. (**E**) The hydrophilic and lipophilic surfaces of BIN/PEI complex after 200 ns MD simulation. (**F**) ITC curves and fitting plots of BIN/PEI. (**G**) The kinect mesostructure of BIN/PEI complex during DPD simulation. (**H**) The mesostructure and cross section (top right corner) of BIN/PEI complex after 20 ns DPD simulation.

We calculated the interaction between BIN and polyethyleneimine (PEI) using the adsorption module of the Materials Studio 2017 (Accelrys Inc, kindly provided by Analytical& Testing Center of Sichuan university) software. The adsorption energy of BIN to PEI was -312.33 ± 2.81 Kcal/mol (Table S1), considerably higher than that of PEI to PEI (Table S2). The lowest energy conformation of the BIN/PEI complex comprised molecular BIN wrapped in a pocket formed by the branch chains and backbone of PEI, stabilized by hydrogen bonds between the carboxyl group of BIN and the amino group of PEI (Figures 1A and B). This was confirmed using Autodock 4.2, which revealed the intermolecular force to be mainly due to H-bonding, electrostatic interactions and van der Waals forces (Figures 1C, S1). The lowest energy conformation of BIN/PEI complex was subjected to 200-ns molecular dynamic (MD) simulation (Figure S2). After MD simulation, BIN was observed to be tightly wrapped in the pocket formed by the branch chains of PEI and aggregated to an amphiphilic complex forming an amphiphilic complex in which PEI and BIN represented the hydrophilic and hydrophobic regions, respectively (Figures 1D and E). The results of computer simulation were verified by characterizing guest-host pairs of BIN and PEI by isothermal titration calorimetry (ITC). The negative enthalpy of the BIN/PEI system indicated an exothermal profile (Figure 1F), suggesting that assembly of the complex was thermodynamically favorable and that the strong interactions between BIN and PEI promote self-assembly of BIN and PEI.

Dissipative particle dynamics (DPD) simulation 30 of the self-assembly of BIN and PEI in water revealed that the various energies of the whole system declined rapidly during the first 1000 ps (Figure S3). During this time, the BIN and PEI mesomolecules gradually changed from a randomly distributed state to small complexes at 100 ps, which then gradually assembled into irregular aggregates (Figure 1G). By 1 ns, the complexes had formed stable spherical aggregates and maintained this structure until 10 or even 20 ns (Figures 1G and H). The cross-section of the final spherical aggregates shows that the BIN beads were evenly distributed among the particles (Figure 1H).

These results strongly suggest that BIN and PEI could self-assemble to nanoparticles in water, from the perspective of atomic scale to mesoscopic scale.

### Preparation and characterization of bindarit-loaded nanoparticles

**Figure 2:**
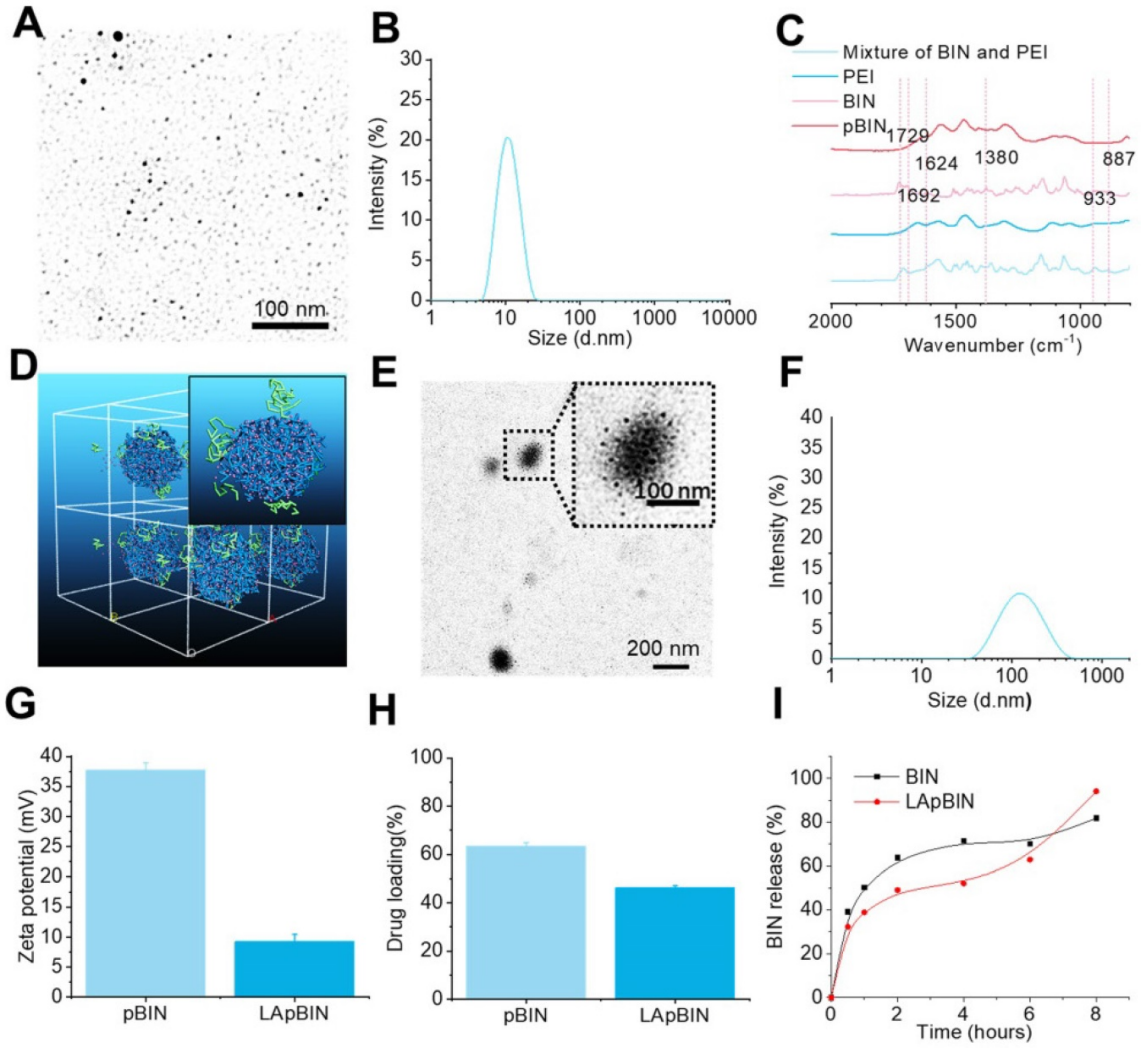
** Characterization of BIN loaded nanoparticles.** (**A, B**) The TEM image (a) and size distribution (B) of pBIN. (**C**) The FTIR spectra of free BIN, PEI and pBIN. (**D**) The mesostructure of LAM absorbed BIN/PEI complex after 20 ns DPD simulation. (**E, F**) The TEM image (**E**) and size distribution (**F**) of LApBIN NPs. (**G-I**) The zeta potential (**G**), drug loading (**H**) and *in vitro* release profiles of raw BIN and LApBIN NPs (**I**).

Guided by the computational simulation, we prepared nanoparticles of BIN and PEI (MW = 25 KDa) (pBIN) with a hydrodynamic radius of 10.4 ± 3.7 nm (Figures 2A and B). Fourier transform infrared spectroscopy (FT-IR) analysis of the forces mediating the self-assembly of BIN and PEI (Figure 2C) revealed that the relatively strong absorption bands at 1624 and 1380 cm^-1^ (corresponding to the asymmetric and symmetric stretching vibrations of anionic carboxyl, respectively) of raw BIN were significantly attenuated when it formed nanoparticles with PEI, suggesting electrostatic interactions occurred. Furthermore, the antisymmetric stretching vibrations at 1729 cm^-1^ and 1624 cm^-1^, respectively, attributable to the carbonyl in BIN was absent in the spectrum of the nanoparticles, mainly due to disruption of the H-bonding between BIN dimers. Evident weakening of the absorbance at 933 and 887 cm^-1^, attributable to arylene groups, indicated the association of hydrophobic moieties. These results suggest that the assembly of PEI and BIN is mediated by multiple noncovalent forces including electrostatic interactions, H-bonding, and hydrophobic interactions. Dispersing the pBIN in various concentrations of Tween 20 did not significantly affect the particle size distribution; however, dissociation of pBIN was observed in high concentrations of NaCl or acetic acid, highlighting that hydrogen bonding and ionic interactions played major roles in the formation of pBIN (Figure S4).

Analysis of the adsorption capacity of LAM to pBIN revealed the energy of adsorption of LAM to BIN/PEI to be -912.77 ± 1.93 Kcal/mol (Table S3, Figure S5), considerably higher than that of BIN to PEI. Furthermore, DPD simulation showed that LAM could adsorb to the surface of pBIN (Figure 2D). Under the computer aided guidance, LAM-coated pBIN nanoparticles (LApBIN) were successfully prepared by adding LAM into the suspension of pBIN. Transmission electron microscopy result revealed that LApBIN were formed by a good deal of small nanoparticles (Figure 2E), with a particle size significantly higher than that of pBIN alone (Figure 2F). The reason for the size change might be aggregation of nanoparticles due to LAM modification (Figure S6). The zeta-potential of LApBIN was significantly lower than that of pBIN alone (Figure 2G), indicating that LApBIN have different surface properties compared to pBIN. The addition of LAM decreased the drug loading and entrapment efficiency relative to pBIN, but these capacities were still high for LApBIN (Figures 2H, S7). In addition, the release of BIN was not significantly affected by adsorption of LAM (Figure 2I).

### *In vitro* activity of bindarit-loaded nanoparticles

**Figure 3:**
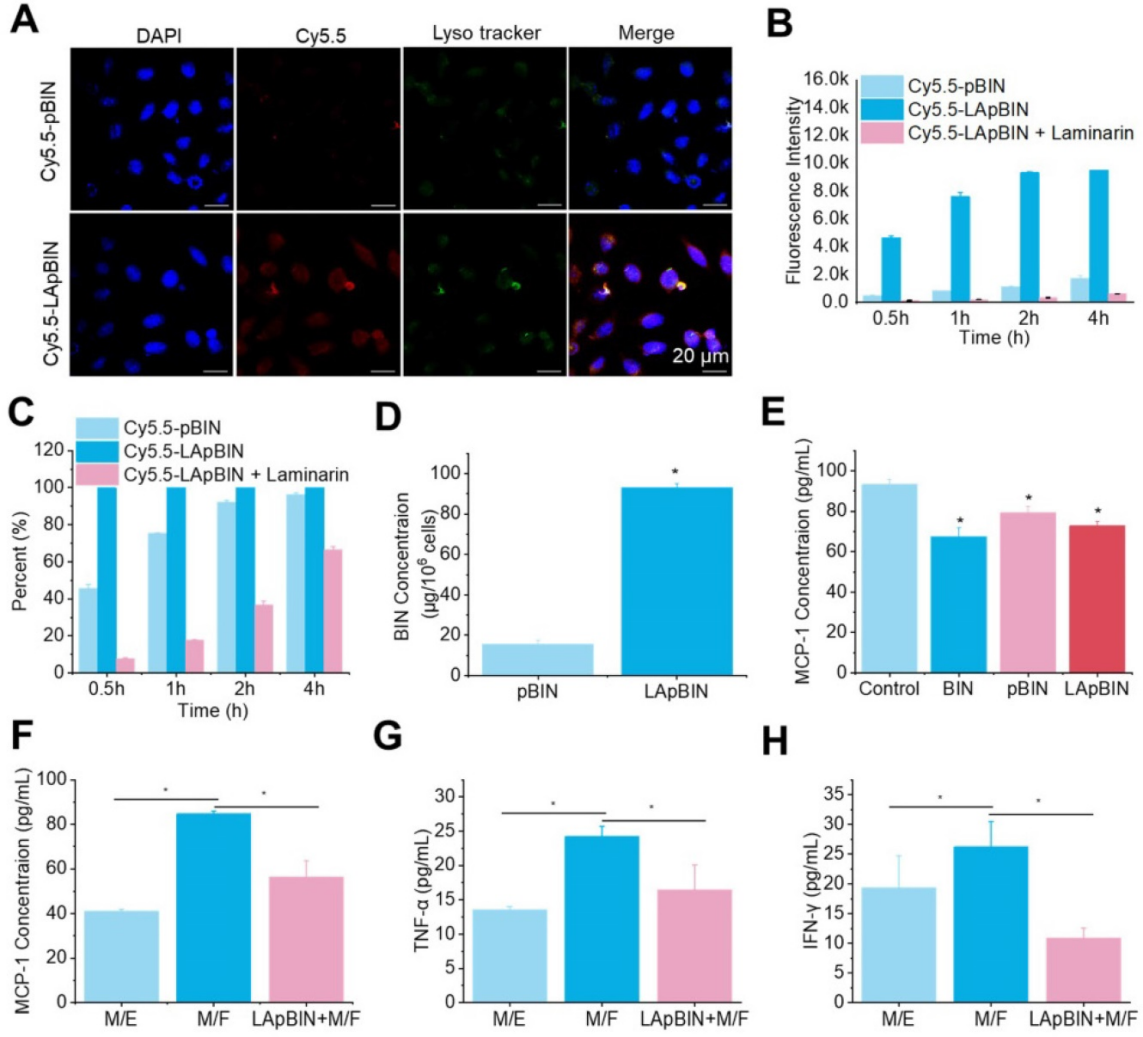
** The* in vitro* activity of BIN loaded nanoparticles.** (**A**) The fluorescence images of Raw 264.7 cells after co-incaution with various Cy5.5-labeled nanoparticles for 4 h. (B-C) Kinect fluorescence intensity (**B**) and Cy5.5 positive cells percent (**C**) in Raw 264.7 cells measured by flow cytometry. (**D**) BIN concentrations in Raw 264.7 cells after 4 h co-incubation with Cy5.5 labeled nanoparticles. (**E**) The concentration of MCP-1 in Raw 264.7 cells culture medium. (**F-H**) The concentrations of MCP-1 (F), TNF-α (G) and INF-γ (H) concentrations in cell culture medium in co-culture systems of macrophages with foam cells (M/F) or with endothelial cells (M/E). n=3, * means p<0.05.

Laminarin is a specific ligand of Dectin-1 receptor 31, which is presented on the surface of monocytes and macrophages 32. Our analysis of Cy5.5-labeled pBIN (Cy5.5-pBIN) and LApBIN (Cy5.5-LApBIN) particles revealed the uptake of Cy5.5-LApBIN by Raw 264.7 cells to be much higher than that of Cy5.5-pBIN (Figures 3A and B); in fact, almost 100% of the Raw 264.7 cells exhibited uptake of LApBIN after incubation with the particles for 30 min, whereas only 45.8% exhibited uptake of pBIN in this time (Figure 3C). To verify whether the increased internalization was influenced by surface modification, free LAM was added to pretreat Raw 264.7 cells before co-incubation with LApBIN for blocking Dectin-1 receptor. Uptake of LApBIN by Raw 264.7 cells pretreated with LAM and the percentage of cells involved in phagocytosis were both significantly reduced, compared to that of LApBIN alone (Figures 3B and C).Accordingly, the concentration of BIN was higher in Raw 264.7 cells incubated with LApBIN than in those incubated with pBIN (Figure 3D); therefore, inhibition of MCP-1 secretion was greater in Raw 264.7 cells incubated with LApBIN compared with those incubated with pBIN (Figure 3E). These results suggested that the surface modification of pBIN with LAM could significantly increase the internalization of them by Raw 264.7 cells and enhance the inhibition of MCP-1 secretion.

For further evaluating whether the LApBIN loaded macrophages could migrate to inflammatory locals and inhibit the secretion of MCP-1 in* vitro*, the co-incubation systems of macrophages with foam cells (M/F) and macrophages with endothelial cells (M/E) were, respectively, employed to simulate normal vascular endothelium and atherosclerotic plaque site. In contrast to healthy vascular endothelium composed of endothelial cells that would not secrete MCP-1 for recruiting monocytes, in inflamed lesions or atherosclerotic, monocytes and other inflammatory cells would be recruited to lesions under the guide of MCP-1 secreted by injured cells, macrophages or foam cells 27. Consistent with this, we found that MCP-1 concentration in M/E system was much lower than that in M/F system (Figure 3F). Although the secretion of MCP-1 by Raw 264.7 cells carrying LApBIN would be inhibited (Figure 3E), the migration of Raw 264.7 cells to foam cells was not restricted by the MCP-1 secreted by themselves, but by the MCP-1 secreted by foam cells. Thus, Raw 264.7 cells which had been pre-treated with LApBIN for 6 h could still successfully transfer to the foam cells layer in the M/F system after co-incubation for 4 hours, but little crystal violet labeled Raw 264.7 cells were observed in endothelial cells layer in the M/E system (Figure S8). In addition, decreased expressions of MCP-1, TNF-α and interferon (IFN)-γ were observed in response to LApBIN in the M/F system compared with the control (Figures 3F-H).

Taken together, our results demonstrate that LAM modification of BIN-loaded nanoparticles could confer monocyte-targeting abilities to the particles, and potentially enable effective delivery of BIN to inflammatory tissues *in vivo* to inhibit progression of inflammation.

### Oral delivery of bindarit to monocytes* in vivo*

**Figure 4:**
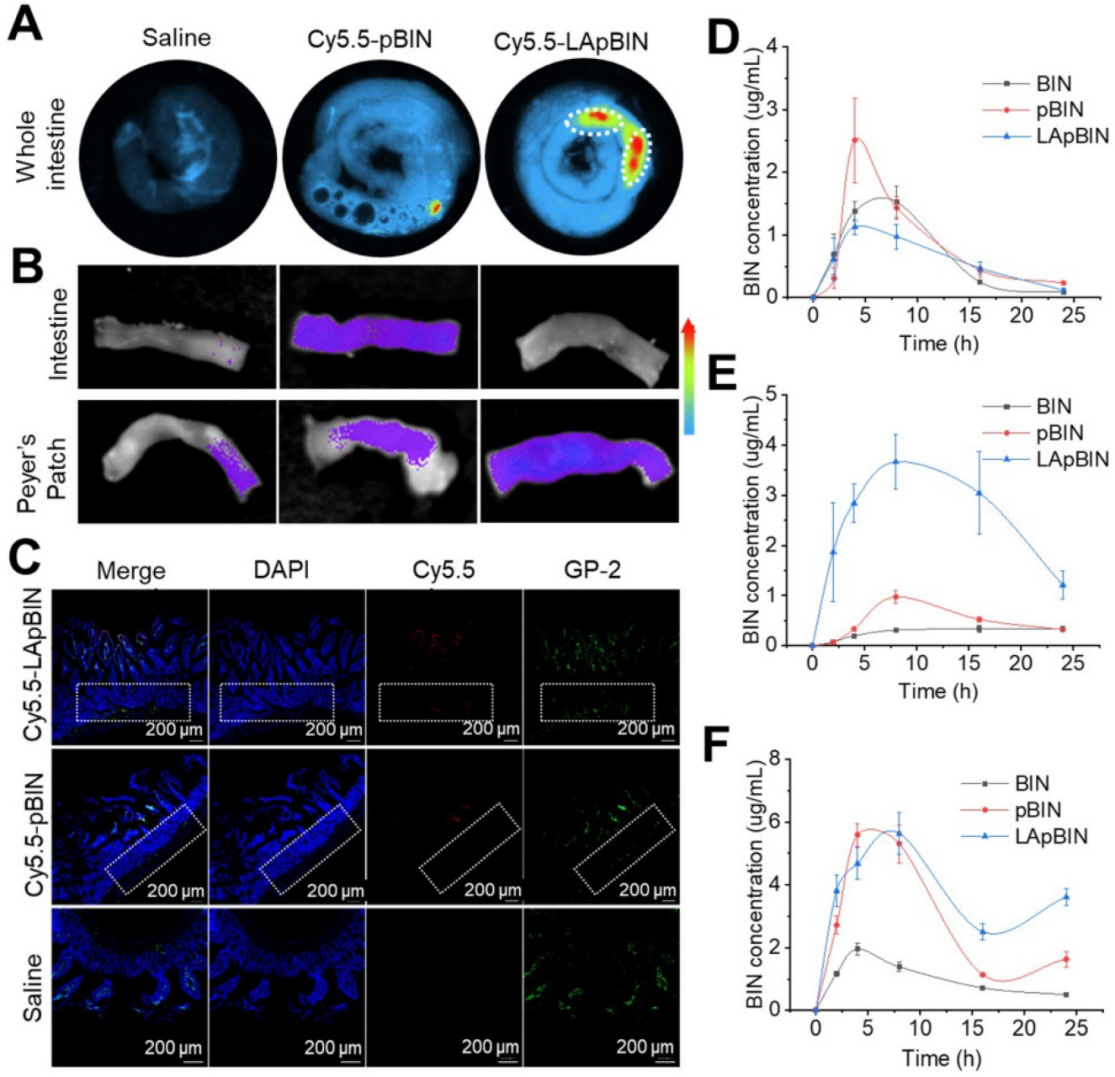
** Oral adsorption of LApBIN though intestinal.** (**A**) *Ex vivo* images of Cy5.5 distribution in gut. (**B**) *Ex vivo* images of Cy5.5 distribution in Peyer Patch region and normal small intestine. (**C**) Immunofluorescence analysis on distribution of Cy5.5 in Peyer Patch region after administration of various Cy5.5 labeled nanoparticles in the intestinal lumen. M cells were stained with anti-GP-2 antibody (green), while nuclei were counterstained with DAPI (blue). Dashed box indicates the submucosa region. (**D-F**) The dynamic BIN concentrations of different formulations in intestine epithelium (D), Peyer Patch (E) and serum (F).

Although *in vitro* experiments confirmed that LApBIN could be specifically recognized by monocytes/macrophages and then delivered to inflammatory lesions *in vitro*, targeted delivery of BIN to peripheral blood monocytes following oral administration is key to achieve oral targeted delivery of BIN to obesity related lesions. To verify it, the absorption and translocation of nanoparticles in the gut was observed by employing Cy5.5 labeled BIN nanoparticles. We found the absorption and translocation of Cy5.5-BIN in the gut to be considerably higher for Cy5.5-LApBIN compared with Cy5.5-pBIN (Figures 4A, S9A) when evaluated 4 h after oral administration to mice. Moreover, the fluorescence was significantly higher in Peyer's Patches-gut-associated lymphoid tissues compared with other intestinal regions (Figure 4A), which was confirmed by observations of an isolated Peyer's Patch and normal small intestine regions (Figures 4B, S9B). In Peyer's Patches region, Cy5.5-LApBIN was mainly distributed in glycoprotein 2 (GP-2)-positive intestinal microfold (M) cells (Figures 4C, S10A) which express high levels of Dectin-1 on their surfaces 33-36. In addition, M cells has been proved to be able to deliver samples of foreign material including nanoparticles from the lumen to lymphocytes, macrophages and dendritic cells enfolded in pockets in the basolateral surfaces of M cells 37-40. Fluorescence in the submucosal region demonstrated that LApBIN successfully penetrate the intestinal absorption barriers (Figure 4C).

Analysis of the concentration of BIN in the intestinal epithelium and Peyer's Patches (Figures 4D and E) supported the fluorescence results (Figure 4B), revealing that the concentration of BIN in intestinal epithelia isolated from LApBIN-treated mice was reduced compared with that from pBIN-treated mice (Figure 4D). The concentration of BIN in Peyer's Patch tissue isolated from LApBIN-treated mice was considerably higher compared with that from pBIN-treated mice (Figure 4E). Serum levels of BIN suggested that both pBIN and LApBIN enhanced the absorption of BIN through the gut following oral administration (Figure 4F). Furthermore, the dynamic concentrations of BIN from in heart, liver, spleen, lung and kidney tissue revealed that administration of LApBIN resulted in improved distribution of BIN in liver and lung tissues (Figure S11). Taken together, these results suggest that LApBIN are efficiently absorbed through M cells in Peyer's Patches.

**Figure 5:**
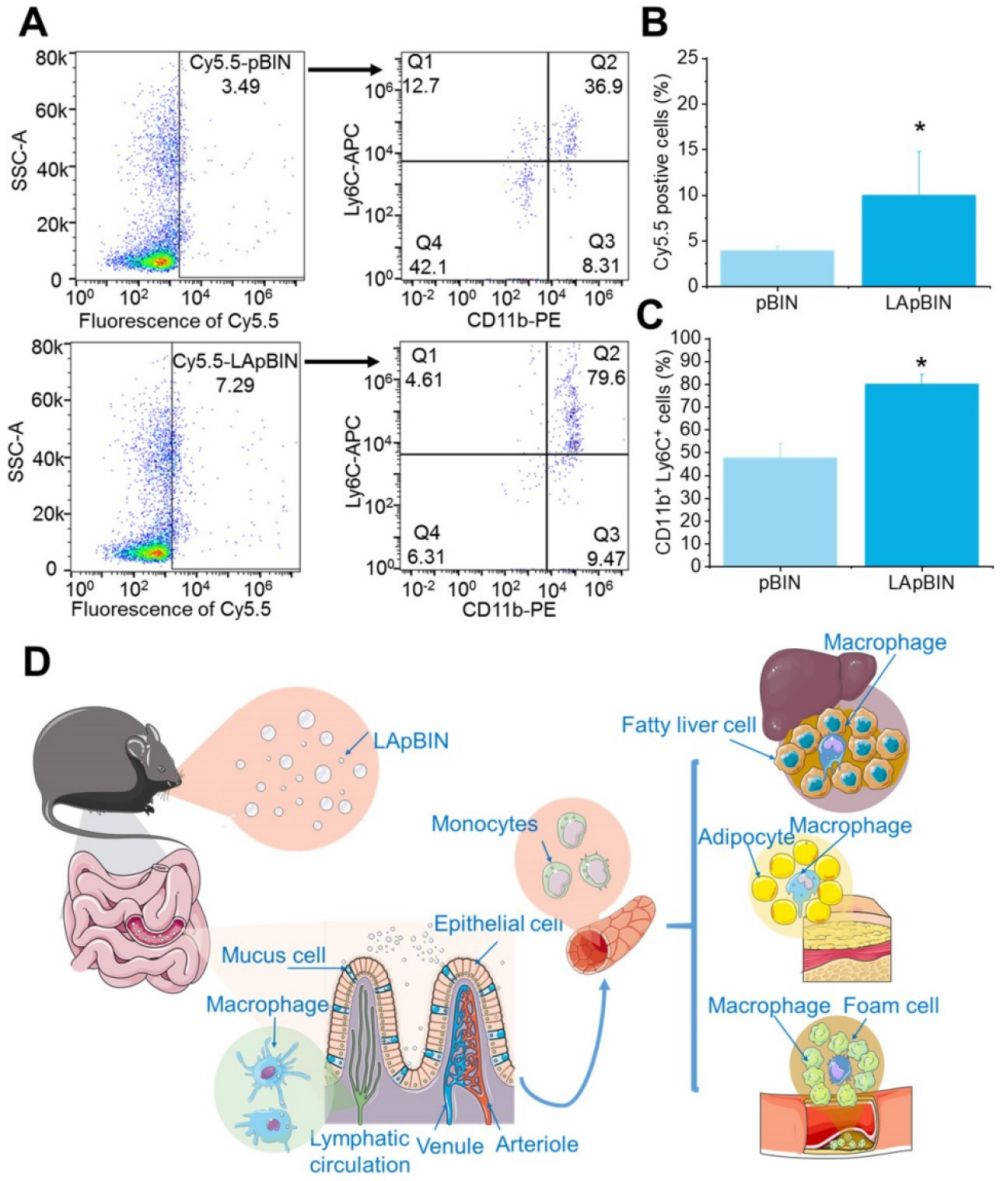
** The distribution of nanoparticles in blood cells.** (**A**) The Cy5.5 distribution in peripheral blood cells detected by flow cytometry. (**B**) The percent of Cy5.5 positive cells in blood cells quantified by flow cytometry. (**C**) The percent of CD11b^+^ Ly6C^+^ cells in Cy5.5 positive cells quantified by flow cytometry. (**D**) Schematic diagram of LAM-mediated oral targeting of nanoparticles to diseased sites of obesity related diseases distant from the gastrointestinal tract.

Our analysis of the uptake of nanoparticles by blood cells revealed the proportion of Cy5.5-positive blood cells to be significantly higher following oral administration of LApBIN compared with pBIN (Figures 5A and B). The proportion of CD11b^+^ Ly6C^+^ cells (Ly6C^+^ monocytes) among the Cy5.5-positive cells (Figures 5A and C) was significantly higher in samples from LApBIN-treated mice compared with pBIN-treated mice (Figures 5A and C). These results demonstrate that LApBIN are absorbed by M cells in intestinal Peyer's Patches region after oral administration, then distributed to Ly6C^+^ monocytes in the peripheral blood (Figure 5D), which is in consistent with previous studies based on yeast derived particles (a water insoluble beta-1,3-glucan) 28, 41, 42. Based on the recruitment of Ly6C^+^ monocytes in obesity related lesions, we believe that these LApBIN-loaded Ly6C monocytes can act as “Trojan horse” to deliver drugs to inflammatory adipose tissue, fatty liver lesions and atherosclerotic plaques and thus deliver targeted therapeutics and inhibit inflammation in these lesions in obesity and ORDs (Figure 5D).

### Targeting therapeutics to low-grade inflammatory adipose tissue

**Figure 6:**
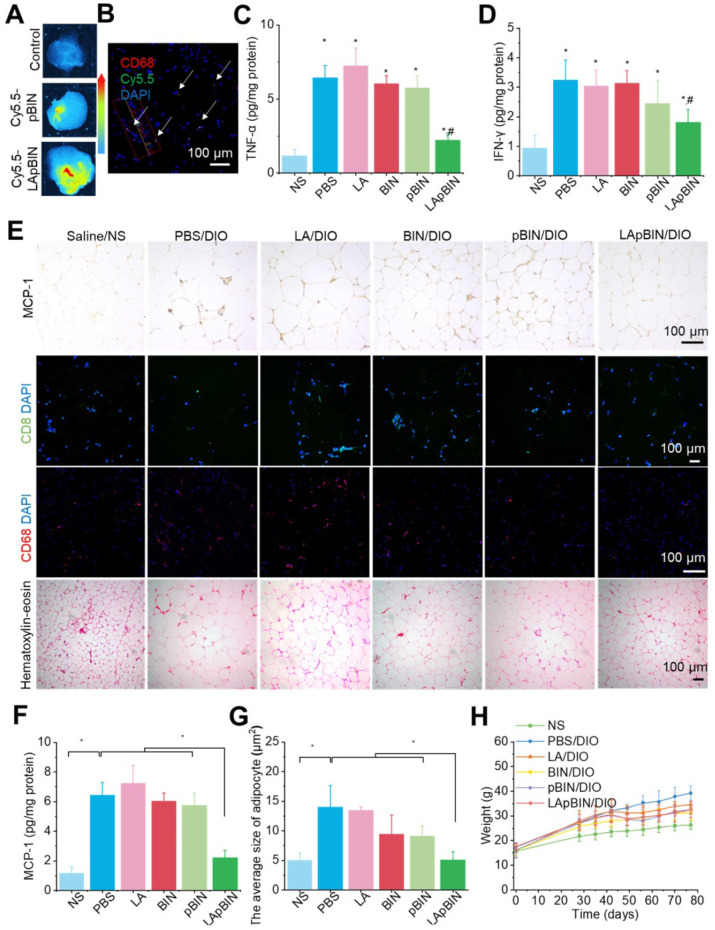
** Targeting ability and therapy effect of BIN loaded nanoparticles in inflammatory inguinal adipose tissues.** (**A**)* Ex vivo* images of Cy5.5 distribution in adipose tissues. (**B**) Co-localization of Cy5.5 (green) and macrophage marker CD68 (red) in adipose tissues isolated from LApBIN treated DIO mice. (**C, D**) The concentrations of TNF-α (C) and IFN-γ (D) in inguinal adipose tissues. (**E**) Histochemistry and H&E staining images of inguinal adipose tissues after various treatments. (**F**) The concentration of MCP-1 in inguinal adipose tissues. (**G**) The average quantified size of adipocytes. (**H**) The changes in weight of DIO mice during various treatments. n=5, * means p<0.05.

The targeting ability of LApBIN to low-grade inflammatory adipose tissue was evaluated using a diet induced obesity (DIO) mouse model, results revealed that recruitment of monocytes in inflammatory adipose tissue resulted in efficient delivery of Cy5.5-LApBIN to inflammatory adipose tissue (Figures 6A, S12), in which fluorescence of Cy5.5 was mainly observed in CD68^+^ macrophages (Figures 6B, S10B, S13). In contrast, little fluorescence was detected in adipose tissue samples from Cy5.5-pBIN-treated DIO mice (Figures 6A, S10B, S13). Low doses of BIN (100 mg/kg once every 3 days) in the form of LApBIN elicited a much stronger anti-inflammatory effect on inflammatory adipose tissue compared with other BIN formulations at same doses of BIN. Secretion of inflammatory cytokines such as TNF-α and interferon-γ (IFN-γ) from adipose tissue was significantly inhibited in DIO mice by oral administration of LApBIN (Figures 6C and D) due to the decreased expression of MCP-1 in adipose tissue (Figures 6E and F, S14A). In addition, the infiltration of inflammatory cells, such as CD68^+^ macrophages and CD8^+^ T cells in adipose tissues were decreased in LApBIN-treated DIO mice (Figures 6E, S14B and S14C). In contrast, administration of free BIN or pBIN did not significant affect the expression of MCP-1, recruitment of inflammatory cells or secretion of inflammatory cytokines (Figures 6C-F, S14). Furthermore, the abnormal growth of adipocytes usually observed in DIO mice 7 was significantly ameliorated by administration of LApBIN (Figures 6E and G). In addition, the food intake, body weight and main blood hematological parameters (except for white blood cell count) were not affected by LApBIN treatment, demonstrating its safety (Figures 6H, S15, S16). The white blood cell count has been reported to be high in DIO mice, possibly due to systemic chronic inflammation caused by obesity 43; we found this to be significantly inhibited by the administration of LApBIN (Figure S16).

Our findings demonstrate that administration of LApBIN achieves targeted delivery of BIN to inflammatory adipose tissue and significantly inhibits the expression of MCP-1, recruitment of inflammatory cells and secretion of inflammatory cytokines in this tissue, and reduces the augment of adipocyte size.

### Therapeutic effect on obesity induced insulin resistance

**Figure 7:**
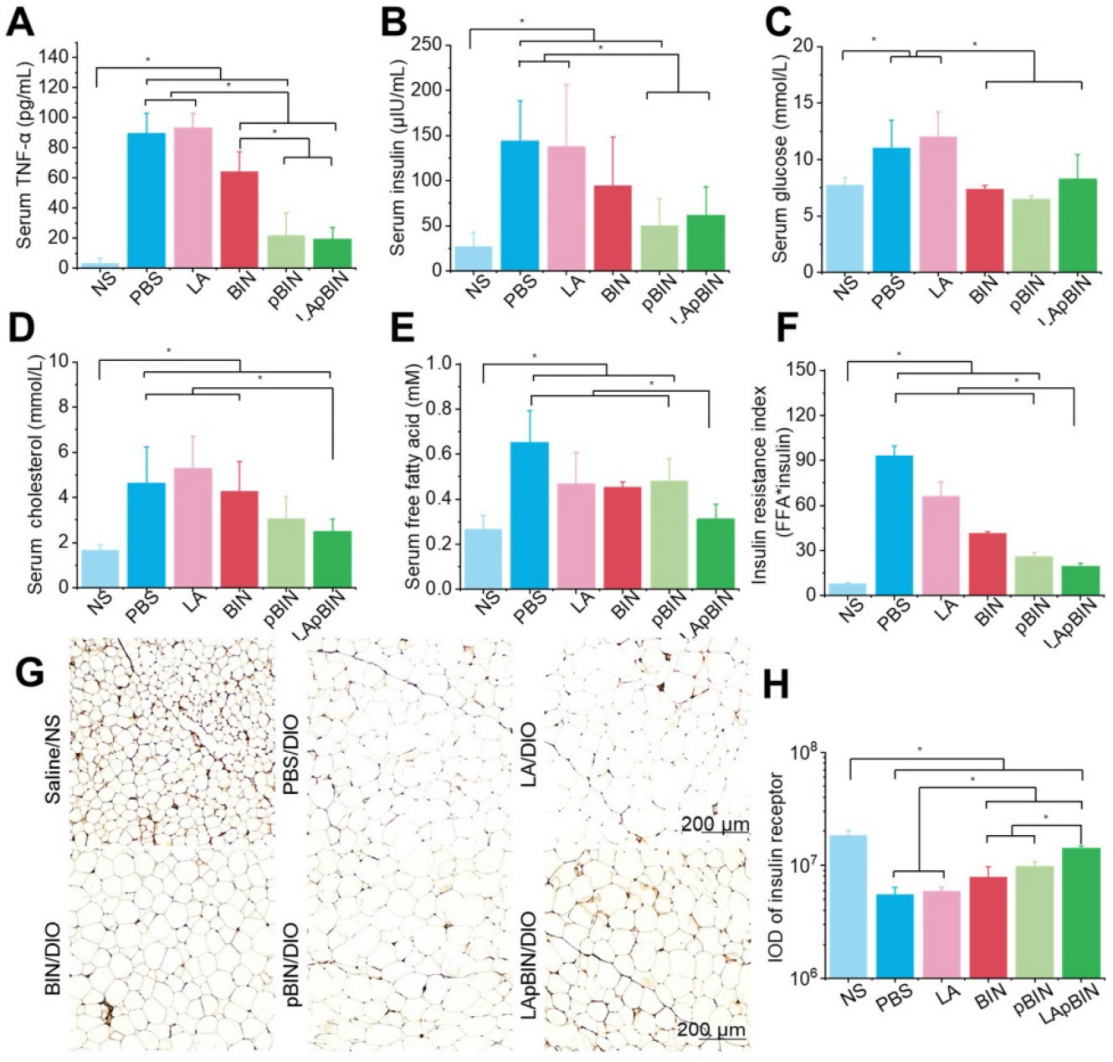
** Therapy effect of BIN loaded nanoparticles in obesity-induced insulin resistance.** The concentrations of serum TNF-α (**A**), insulin (**B**), blood glucose (**C**), cholesterol (**D**), free fatty acid (**E**) in DIO mice treated with various BIN formulations. (**F**) The insulin resistance index according to the concentration of free fatty acid (FFA) and insulin. (**G**) The insulin receptor staining images of inguinal adipose tissues after various treatments. (**H**) The quantified integral optical density (IOD) of insulin receptors in adipose tissue. n=5, * means p<0.05.

Low-grade chronic systemic inflammation is an important cause of insulin resistance 44. It is well known that the increased TNF-α is an important inflammatory factor and increased serum levels are associated with systemic insulin resistance 45. We found that serum TNF-α was significantly lower in mice treated with BIN formulations compared with DIO mice treated with PBS (Figure 7A), and that this effect was more pronounced following administration of pBIN and LApBIN compared with free BIN (Figure 7A). This may be a result of the increased serum concentration of BIN (Figure 4F). Thus, the increase of serum insulin due to negative regulation of insulin resistance in DIO mice was significantly inhibited by pBIN and LApBIN (Figure 7B), and blood glucose was controlled to a level similar to that of C57/BL mice who received a normal diet (Figure 7C). In addition, blood lipid concentration of DIO mice was normalized to varying degrees following treatment with various BIN formulations. Free BIN and pBIN respectively caused the concentration of serum triglyceride and cholesterol to decrease, while LApBIN caused the concentrations of both serum triglyceride and cholesterol to decrease (Figures 7D, S17). Additionally, the concentrations of free fatty acid in serum of DIO mice treated with various BIN formulations was significantly lower compared with that of DIO mice treated with PBS (Figure 7E). The insulin resistance index of LApBIN treated DIO mice was significantly lower than that of other DIO mice (Figure 7F).

Although both pBIN and LApBIN suppressed serum TNF-α levels (Figure 7A), LApBIN treatment resulted in a lower insulin resistance index than pBIN treatment (Figure 7F). This may be due to the regulatory effect of LApBIN on inflammation in adipose tissue, leading to reduced expression of the insulin receptor 45. Accordingly, expression of the insulin receptor in adipose tissues was significantly decreased in DIO mice treated with PBS compared with normal mice. DIO mice who were treated with BIN formulations exhibited significantly enhanced expression of insulin receptor in adipose tissue, with LApBIN exerting the greatest effects to this end (Figures 7G and H). This may be attributable to the lower concentration of inflammatory cytokines in adipose tissue from LApBIN treated DIO mice (Figure 6).

These results demonstrate that LApBIN effectively improve obesity-induced insulin resistance by reducing both systemic inflammation and adipose inflammation.

### Targeting therapeutic of fatty liver

**Figure 8:**
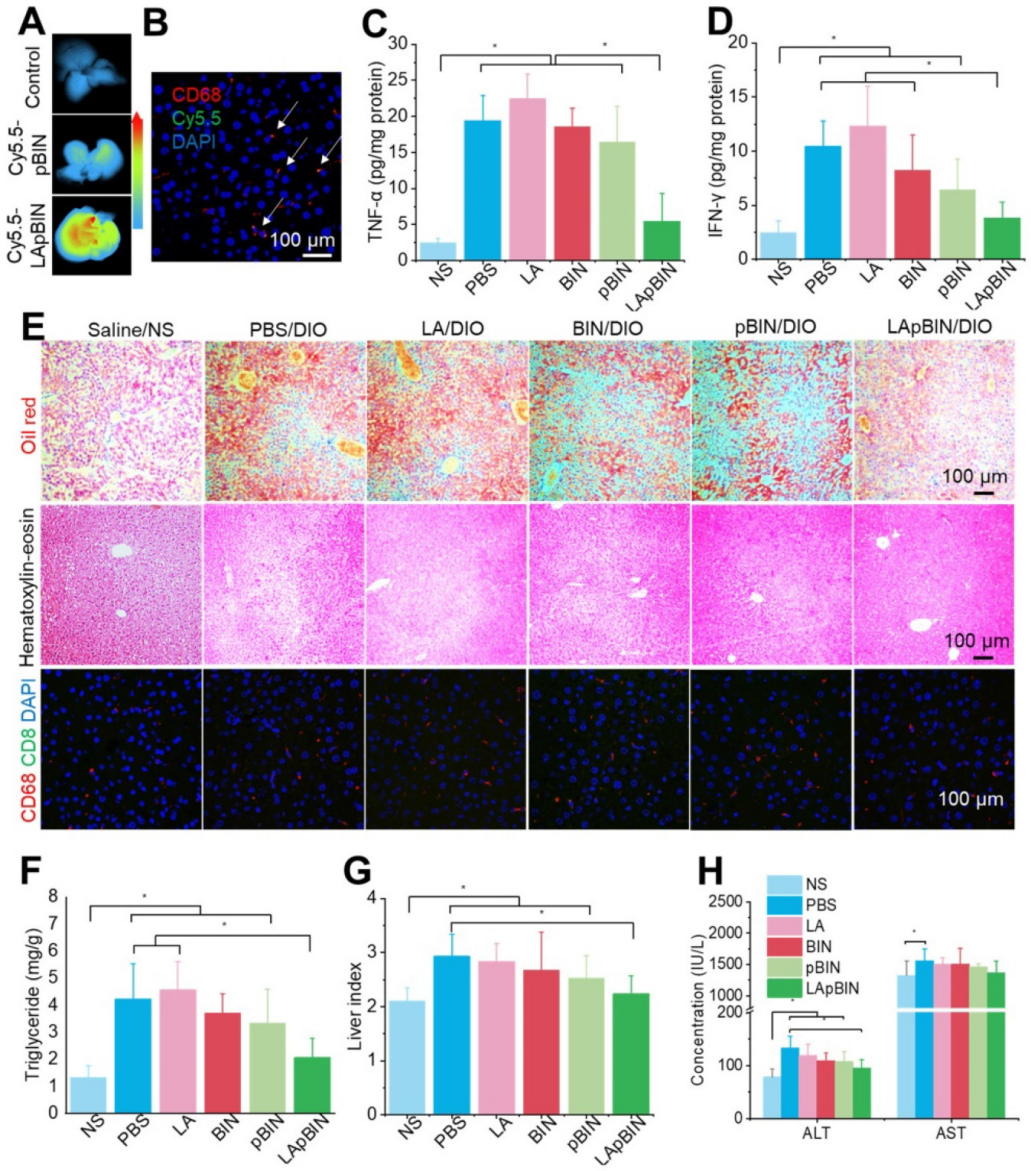
** Targeting ability and therapy effect of BIN loaded nanoparticles in fatty liver tissues.** (**A**)* Ex vivo* images of Cy5.5 distribution in fatty liver tissues. (**B**) Co-localization of Cy5.5 (green) and macrophage marker CD68 (red) in fatty liver tissues isolated from LApBIN treated DIO mice. (**C, D**) The concentrations of TNF-α (C) and IFN-γ (D) in fatty liver tissues. (**E**) Histochemistry, oil red staining, and hematoxylin & eosin staining images of fatty liver tissues after various treatments. (**F**) The liver index of DIO mice after various treatments. (**G**) The concentration of triglyceride in liver after various treatments. (**H**) The concentrations of alanine aminotransferase (ALT) and aspartate aminotransferase (AST) in blood. n=5, * means p<0.05.

Along with the recruitment of monocytes to inflammatory adipose tissue, large numbers of monocytes are recruited to the fatty liver during the progression of fatty liver and fibrosis 46. Thus, compared with the DIO mice treated with Cy5.5-pBIN, stronger fluorescence of Cy5.5 was observed in fatty liver lesions of DIO mice treated with Cy5.5-LApBIN (Figures 8A, S18), and mainly distributed in CD68^+^ macrophages in liver tissues following oral administration (Figures 8B, S19, S10C). Compared with Cy5.5-pBIN, the Cy5.5 fluorescence signals in heart and kidney tissues of Cy5.5-LApBIN treated DIO mice were higher (Figure S20). It is noting that the fluorescence signal of Cy5.5 in lung tissue of Cy5.5-LApBIN treated DIO mice was lower than that of pBIN treated DIO mice (Figure S20), which is not in consistent with the previous BIN concentration determined in lung tissues (Figure S11). This difference may be derived from the different mice models and different administration times employed in the experiments evaluating fluorescence and drug distribution. These results suggested that Cy5.5-LApBIN were successfully delivered to fatty liver.

Due to the targeting ability of LApBIN to fatty liver lesions, expression of pro-inflammatory cytokines including TNF-α (Figure 8C) and IFN-γ (Figure 8D) and number of CD68+ Kupffer cells/macrophages (Figures 8E, S21) were considerably reduced in liver tissues of DIO mice treated with LApBIN compared with other formulations. Due to reduction of inflammation, the lipid stained by Oil Red O (Figures 8E, S21) and triglyceride concentration (Figure 8F) in liver tissues of LApBIN-treated DIO mice were greatly reduced compared with DIO mice who received other treatments. In addition, representative hematoxylin & eosin staining images suggested a reduced formation of vacuoles in liver cells of LApBIN treated DIO mice (Figure 8E). The regulation of inflammation by LApBIN in the case of fatty liver resulted in considerable alleviation of symptoms; primarily, normalization of liver index and function, compared with mice who received a normal diet (Figures 8G and H).

These results demonstrate that LApBIN can be effectively delivered to fatty liver and exert protective effects by reducing fatty liver weight, suppressing the secretion of inflammation-associated cytokines, reducing the lipid accumulation in liver cells and restoring normal liver function.

### Targeting therapeutic of atherosclerosis

**Figure 9:**
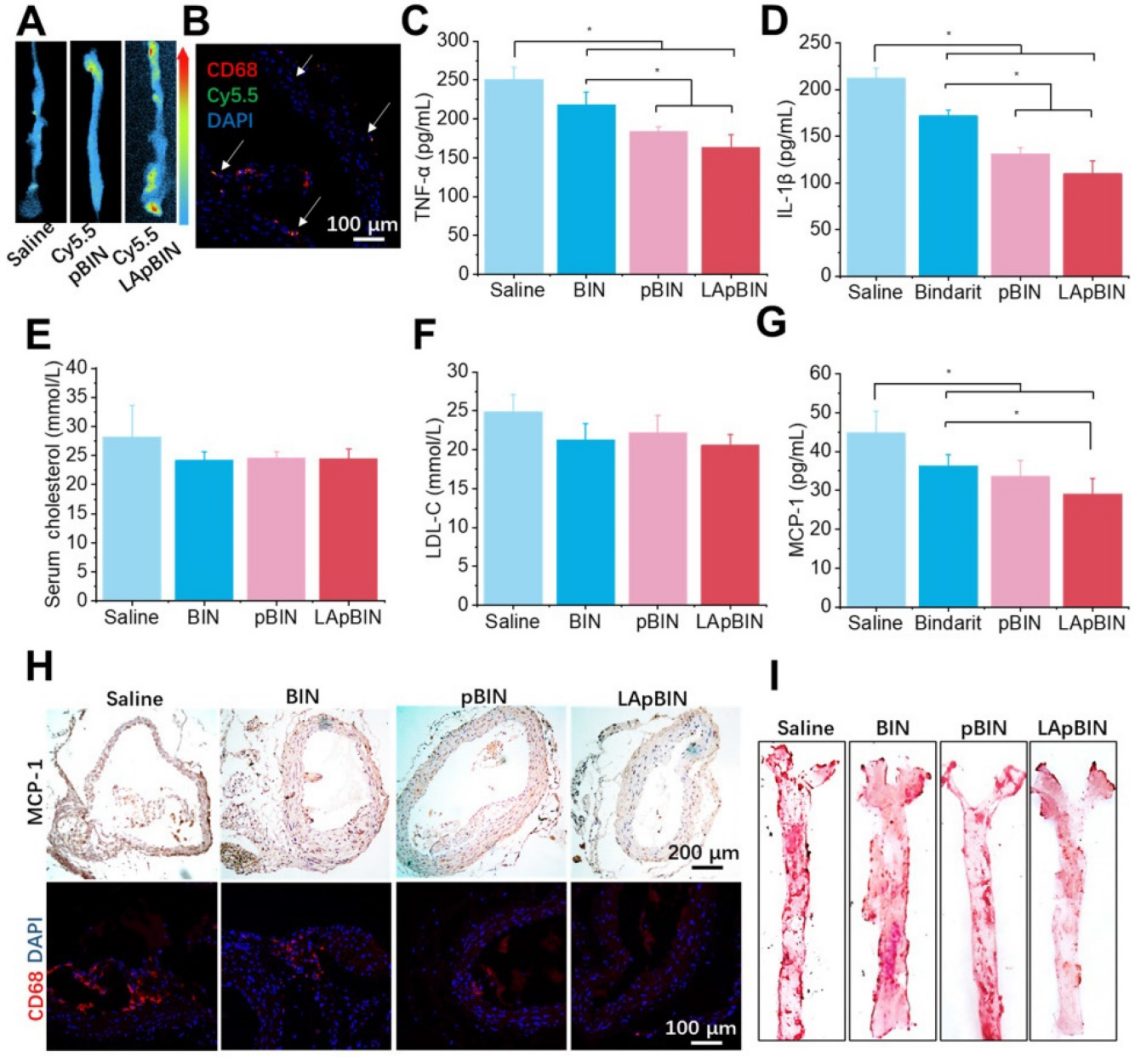
Targeting ability and therapy effect of BIN loaded nanoparticles to atherosclerosis plaques in ApoE**^-^**mice. (A)* Ex vivo* images of Cy5.5 distribution in aortic tissues. (B) Co-localization of Cy5.5 (green) and macrophage marker CD68 (red) in aortic tissues isolated from LApBIN treated ApoE**^-^**mice. (C, D) The concentrations of serum TNF-α (C), IL-1β (D), cholesterol (E), low density lipoprotein (LDL) (F) and MCP-1(G) in ApoE**^-^**mice treated with various BIN formulations. (H) The expression of MCP-1 and CD68 in atherosclerosis plaques. (I) The oil-red staining images of aortic tissues isolated from ApoE**^-^**mice. n=5, * means p<0.05.

Induction of atherosclerosis in wild-type mice is difficult using a high-fat diet; thus, we used ApoE knockout C57/BL mice (ApoE**^-^**mice) to build the atherosclerosis model 47, which was induced by feeding a high-cholesterol/fat diet. Contributed by recruitment of monocytes to plaques in ApoE**^-^**mice, targeting of Cy5.5-LApBIN to atherosclerotic plaques was successfully achieved by oral administration (Figures 9A, S22). The Cy5.5-labled nanoparticles were mainly distributed in CD68^+^ macrophages (Figures 9B, S10D), while little fluorescence was detected in plaques following administration of Cy5.5-pBIN (Figures S23, S10D). In the fatty liver of DIO mice, administration of Cy5.5-LApBIN increased the fluorescence signal of Cy5.5 in the liver. In contrast, fluorescence was lower in liver tissues of ApoE**^-^**mice treated with Cy5.5-LApBIN compared with Cy5.5-pBIN (Figure S24), which may be attributable to the absence of inflammation and consequent reduction in monocytes recruitment to the liver of ApoE**^-^**mice.

The administration of various BIN formulations to ApoE**^-^**mice previously fed with a high-cholesterol/fat diet for 4 weeks had no influence on the weight of ApoE**^-^**mice and no significant side effects were observed (Figure S25). Hematoxylin and eosin staining revealed no pathological changes in any of the organ sections (Figure S26). Low-grade systemic inflammation can cause abnormal lipid metabolism, leading to the development of atherosclerosis 48-50. Our results demonstrate the efficacy of LApBIN for the treatment of atherosclerosis, although administration of all BIN formulations caused effective reduction of TNF-α (Figure 9C) and IL-1β (Figure 9D) concentrations in peripheral blood, LApBIN were associated with largest reduction of these pro-inflammatory cytokines. ApoE**^-^**mice treated with free BIN, pBIN and LApBIN all had lower serum total cholesterol (Figure 9E) and low-density lipoprotein compared with those treated with PBS (Figure 9F), although these differences were not statistically significant. Administration of BIN formulations had little effect on the concentration of triglycerides or high-density lipoprotein (Figure S27). In addition to its effects on lipid metabolism through improvement of systemic inflammation, LApBIN reduce the expression of MCP-1 in peripheral blood and local plaques, leading to reduced aggregation of CD68^+^ macrophages in plaques (Figures 9G and H, S28). Through these effects, LApBIN significantly inhibited the formation of atherosclerotic plaques (Figures 9I, S29).

Our findings indicate that LApBIN reduce the level of MCP-1 in sera and plaques by effectively delivering BIN to atherosclerotic plaques and inhibiting the accumulation of macrophages in plaques. Through the regulation of local inflammation in plaques, LApBIN significantly reduce the formation of atherosclerotic plaques in the aortic arch, thus representing an excellent targeted approach for the prevention of atherosclerosis.

## Conclusions

We present our successful design of LAM-modified BIN-loaded nanoparticles through computer simulation and describe their facile preparation. Our investigations with mouse models of diet-induced obesity and atherosclerosis demonstrate that these LAM-modified nanoparticles can act as effective carriers for bindarit, achieving effective targeting of oral drugs to multiple lesions associated with obesity including inflammatory adipose tissue, fatty liver injury and atherosclerosis. This targeting effect is mainly attributable to the presence of LAM on the particle surfaces, and is achieved by exploiting circulating-monocyte-mediated translocation. However, further studies are required to elucidate the route of LApBIN translocation from intestinal lymph tissue to peripheral blood monocytes, and to clarify whether other mechanisms such as nanoscale-mediated passive targeting or direct migration of LApBIN-loaded lymphatic macrophages are involved in this targeted translocation. This targeting effect, in addition to the regulation of lipid metabolism and insulin resistance through inhibition of low-intensity chronic systemic inflammation, means that oral administration of LApBIN achieves comprehensive regulation of obesity and obesity-related diseases concomitant reduction of inflammation of adipose tissue, reduction adipocyte size, improvement of insulin resistance and prevention of the development of fatty liver and atherosclerotic plaques. More importantly, this strategy of oral targeted therapies for various lesions associated with obesity and ODRs provides a promising approach for the development of effective oral treatments for obesity and other metabolic diseases.

## Methods

### Computational Simulation

We used a multiscale simulation method incorporating atomistic simulation and DPD 30, 51, 52. The three-dimensional (3D) atomistic structure of BIN was download from Drugbank.com, while those of LAM and PEI was sketched using Materials Studio 2017. All atomistic structures were optimized through two cycles of geometry optimization, annealing and quenching in the Forcite module of the software. The energy of adsorption of BIN to PEI and of BIN to the BIN/PEI complex was simulated using the Adsorption module. The stability of the BIN/PEI complex was evaluated through 200-ns molecular dynamics using the Forcite module. Prior to DPD simulation, BIN, PEI, LAM and water were coarse-grained to the appropriate beads (Figure S30). The Flory-Huggins interaction parameters were calculated between beads using the Blends module (Table S3) then DPD simulation was used to investigate the assembly of the nanoparticles. We performed ITC experiments using an iTC200 microcalorimeter (MicroCal Inc., GE Healthcare, USA) operated at 25 °C. Detailed simulation parameters are provided in the Supplementary materials.

### Fabrication of bindarit-loaded nanoparticles

We used a dialysis procedure described in our previous study 53-55 to prepare pBIN. Briefly, 5 mg BIN, and 5 mg PEI were dissolved in 1 mL dimethyl sulfoxide then dialyzed against deionized water at 25 °C. The deionized water was exchanged every 2 h. After 12 h of dialysis, pBIN were purified by collecting the solution in the dialysis bag without further treatment. To fabricate LApBIN, 0.1 mL of LAM aqueous solution (10 mg/mL) was added into 1 mL of pBIN suspension (1 mg/mL) then incubated for 1 min with ultrasonic vibration. Free LAM was removed by centrifuging the solution at high speed (12000 RPM) then washing the pellet twice with water. The drug content of the nanoparticles was quantified by high-performance liquid chromatography ((HPLC; LC-20A, Shimadzu, C8 column 4.6 × 150 mm. Mobile phase A was water with 0.01% trifluoroacetic acid, mobile phase B was acetonitrile with 0.01% run at a gradient of 5% to 95% B within 1.3 min at a flow rate was 1 mL/min. Absorption at 294 nm was monitored and the analysis time was 15 min. Drug content was calculated according to Equation 1:


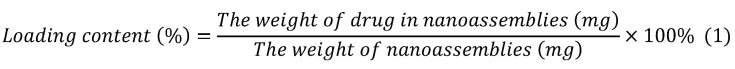


### Elucidation of the self-assembly mechanism of bindarit-loaded nanoparticles

We obtained FT-IR spectra of raw BIN, PEI, BIN/PEI mixture and pBIN and investigated the effects of non-covalent interactions such as hydrophobic interactions, hydrogen bonding and ionic interactions using reported methods 56, 57. Briefly, Tween 20, NaCl or acetic acid were added to pBIN solutions to final concentrations of 1, 5, 10, 20, 50 or 100 mM. After incubation for 30 min, particle size was evaluated by dynamic light scattering (Zetasizer Nano ZS, Malvern Panalytical, United Kingdom).

### Internalization of Cy5.5-labeled nanoparticles by macrophages

Internalization of Cy5.5-labeled nanoparticles by Raw 264.7 cells was monitored by seeding cells into 6-well plates (3×10^5^ cells/plate) then incubating with 3 mL of culture medium for 12 h. After this, 100 μL pBIN (100 μg/mL) or LApBIN (117 μg/mL, to produce the same fluorescence intensity as pBIN) was added and cells were incubated at 37 °C. Phagocytosis was quantified after 0.5, 1, 2 and 4 h by flow cytometry using the FACS Aria II flow cytometer (BD, USA) after the macrophages had been washed and detached. The median fluorescence intensity of the cell population in the APC-Cy7 channel was recorded. In addition, macrophages incubated with various nanoparticles were collected after 4 h of incubation and observed using a Zeiss LSM 710 laser-scanning microscope (Carl Zeiss, Germany).

### Animals

All animal care and experimental protocols were performed under approval from the Animal Ethical and Experimental Committee of the Third Military Medical University (Chongqing, China). Male C57/BL mice (6 weeks old) were obtained from the Animal Center of the Third Military Medical University. All animals were housed in a specific-pathogen-free animal room and fed with a 60% fat diet (high-fat diet; D12492, purchased from OpenSource Diets) for four weeks to induce obesity in C57/BL mice. Once established, DIO mice were fed a high-fat diet throughout the experiment. To establish atherosclerosis, ApoE**^-^**mice, obtained from the Animal Center of the Third Military Medical University, were fed with a normal diet containing 0.25 % cholesterol and 2 % lard for 2 months.

### Oral transport of Cy5.5 labeled nanoparticles

Fifteen DIO mice were orally administered with Cy5.5-pBIN or Cy5.5-LApBIN (BIN dose: 100 mg/kg), 4 h after administration, Cy5.5 fluorescence in intestinal tissues was determined at defined time points. Tissue samples were fixed in 4% (wt/vol) paraformaldehyde at 4°C overnight. For histological assessment, whole-mount staining was performed or tissues were transferred into phosphate-buffered saline (PBS) then embedded in paraffin.

### Cell distribution of Cy5.5-labeled nanoparticles in blood

Nine DIO mice were divided into a control group (one mouse) and two experimental groups (Cy5.5-pBIN [n = 4] or Cy5.5-LApBIN [n = 4]). Mice received Cy5.5-pBIN or Cy5.5-LApBIN (BIN dose at 100 mg/kg) by oral administration, and 100 μL of blood was collected by mandibular sampling 8 h after administration of drugs. Blood was collected into tubes coating heparin sodium, then 600 μL of red blood cell lysis buffer was added and the samples were incubated at 37°C for 10 min. Cells were collected by centrifugation at 400 g for 5 min, then washed three times with PBS and re-suspended in 200 μL of cold PBS and incubated with anti-CD11b and anti-Ly6C antibodies for flow cytometry analysis on the Accuri C6 flow cytometer.

### Biodistribution of nanoparticles in diet-induced obesity and ApoE^-^ mouse models

We divided 12 DIO mice and 12 ApoE**^-^**mice with atherosclerosis into three groups to receive daily oral administration of PBS (control group), Cy5.5-pBIN or Cy5.5-LApBIN (BIN dose of 100 mg/kg) for 4 days. 8 h after the last administration of Cy5.5-labeled nanoparticles, mice were sacrificed, and various tissues including inguinal adipose tissue, aortas, liver, spleen, kidney and thymus were resected for *ex vivo* imaging using a living imaging system (IVIS Spectrum, PerkinElmer).

### Tissue distribution of bindarit in mice

Twelve C57/BL mice were divided to three group (n=4), and respectively received BIN, pBIN and LApBIN (BIN dose of 100 mg/kg of BIN) via oral administration once. At specified time points, animals were euthanized. Blood was collected and organs were excised and weighed. Tissues were homogenized with saline, and the BIN concentrations in the blood and different organs were quantified by HPLC.

### Efficacy of bindarit treatment for obesity and obesity related diseases

Twenty five DIO mice were randomly divided into five groups to receive oral administration of PBS, LAM, BIN, pBIN or LApBIN every 3 days (BIN dose of 100 mg/kg of BIN) for 8 weeks. Five C57/BL mice were used as normal controls fed with normal diet and received oral administration of saline. Twenty male ApoE**^-^**mice were randomly divided into four groups to receive oral administration of PBS, BIN, pBIN or LApBIN (BIN dose of 100 mg/kg) every 3 days for 4 weeks, after which mice were euthanized. Blood was collected and inguinal adipose tissue, whole aortas, aortic sinuses and main organs were harvested to assess the therapeutic efficacy and incidence of adverse effects of the various treatments.

### Quantification of atherosclerotic plaques

We quantified the extent of pathological changes in ApoE**^-^**mice after euthanasia by measuring the areas of lesions in the aorta between the heart and iliac bifurcation. Briefly, aorta samples were fixed by perfusion with formalin (10 % in PBS) for 50 min, after which the peri-adventitial tissue was cleaned, and the aorta was opened longitudinally and stained with Oil Red O stain to evaluate plaque areas.

### Histology and immunohistochemistry analyses

For immunohistochemistry, 6-μm sections were deparaffinized and dried at 60°C. The activity of endogenous peroxidase was inhibited by incubation in 3% hydrogen peroxide and methanol for 20 min, and sections were blocked in PBS containing 1% bovine serum albumin (BSA) and 0.3% Triton X-100 for 60 min. Subsequently, sections were incubated with antibodies to GP-2 (to identify M cells), CD68 (to identify macrophages), CD8 (to identify T cells) insulin receptor and MCP-1.

### Measurement of inflammatory cytokines in serum

Blood and tissue samples were collected after the relevant treatments. The concentrations of typical inflammatory cytokines including TNF-α and INF-γ were determined using enzyme-linked immunoassay kits (Huijia Biotechnology Co., China) according to the manufacturer's protocols.

### Assessments of serological parameters

Serum levels of free fatty acid, glucose, triglycerides, low- and high-density lipoprotein, and cholesterol from fasted obese mice were measured by standard kits from Abcam (USA). Serum insulin was determined from samples taken from fasted obese mice by ELISA assay performed according to manufacturer's protocol (Huijia Biotechnology Co., China).

### Statistical and image analysis

The Predictive Analytics Software 18 package was used for all statistical analyses. After evaluating the homogeneity of data, independent continuous variables were evaluated using analysis of variance analysis (ANOVA). One-way ANOVA was used to compare more than two groups, while an unpaired t-test was used for comparisons between two groups. All data are expressed as mean ± standard deviation (SD), and significance was accepted at p < 0.05. Quantitative evaluations of stained section samples were performed using Image-Pro Plus 6.0 software.

## Supplementary Material

Supplementary experimental section, figures, and tables.

## Abbreviations

ORD: obesity related disease
TNF-α: tumor necrosis factor
IL1-β: interleukin 1-β
MCP-1: monocyte chemoattractant protein-1
BIN: bindarit
CCL2: C-C motif chemokine ligand 2
LAM: laminarin
PEI: polyethyleneimine
MS: Materials Studio
MD: molecular dynamic
DPD: dissipative particle dynamics
MW: molecular weight
pBIN: BIN/PEI nanoparticles
LApBIN: LAM coated pBIN nanoparticles
FT-IR: Fourier transform infrared spectroscopy
M/F: macrophages with foam cells
M/E: macrophages with endothelial cells
GP-2: Glycoprotein 2
M cells: microfold cells
DIO: diet induced obesity
IFN-γ: interferon-γ
H&E: Hematoxylin & Eosin staining
ApoE-/mice: ApoE knocked C57/BL mice
WBC: white blood cells
LDL: low density lipoprotein
HDL: high density lipoprotein
HPLC: high performance liquid chromatography
Cy5.5-pBIN: Cy5.5 labeled pBIN
Cy5.5-LApBIN: Cy5.5 labeled LApBIN
PFA: paraformaldehyde
SD: standard deviation
