# Supplementary material for: Prevention of Obesity Related Diseases through Laminarin-induced targeted delivery of Bindarit
Source: Theranostics. 2020 Jul 25;10(21):9544–60. doi: 10.7150/thno.45788 (PMC7449909; doi:10.7150/thno.45788)
Supplement: Supplementary file 1 — Supplementary experimental section, figures, and tables. [file thnov10p9544s1.pdf]

Supplementary materials

Prevention of Obesity-Related Diseases through Laminarin-Induced Targeted Delivery of Bindarit

Chunmei Xu[1,a], Luqi Yin[2,a], Zhipeng Teng[3,a], Xuemei Zhou[1], Wenjie Li[1], Qiong Lai[1], Cuiping Peng[2], Chengyuan Zhang[1,2], Jie Lou[1]*, Xing Zhou[1,2]*

1. School of Pharmacy and Bioengineering, Chongqing University of Technology, Chongqing 400054, China
2. Department of Pharmaceutics, College of Pharmacy, Third Military Medical University, Chongqing 400038, China
3. Department of Neurosurgery, Chongqing Traditional Chinese Medicine Hospital, Chongqing 400021, China;
a. These authors contribute equally to this paper.

Corresponding authors:
Xing Zhou, PhD, Prof.
School of Pharmacy and Bioengineering, Chongqing University of Technology, Chongqing 400054, China
Department of Pharmaceutics, College of Pharmacy, Third Military Medical University, Chongqing 400038, China
E-mail: diszhou@126.com & diszhou@cqut.edu.cn

Jie Lou, PhD, Prof.
School of Pharmacy and Bioengineering, Chongqing University of Technology, Chongqing 400054, China
E-mail: loujie@cqut.edu.cn

Supplementary experimental section

Materials

Branched polyethyleneimine (Mw=25000, PEI) and Laminarin (LAM) were obtained from Sigma-Aldrich (St. Louis, MO, USA). Bindarit (BIN) was purchased from Chemlin (Nanjing, China). Rabbit anti-mouse CD68, MCP-1, and CD8 primary antibodies were purchased from Servicebio (Wuhan, China). Rabbit anti-mouse GP-2 primary antibodies were purchased from Bioss (Beijing, China). All fluorescent secondary antibodies for immunostaining were from Servicebio (Wuhan, China). H&E staining kit, oil red dye and hematoxylin dye were purchased from Servicebio (Wuhan, China). Cy5.5 NHS ester was purchased from Lumiprobe (Hallandale Beach, FL, USA). Penicillin, streptomycin, fetal bovine serum (FBS), and Eagle's minimum essential (EME) medium were purchased from HyClone (Waltham, MA, USA). RPMI1640 medium was obtained from Gibco (Grand Island, NY, USA). The high fat diet (D12492) was purchased from OpenSource Diets (Research Diets, New Brunswick, NJ).All the other reagents are commercially available and used as received.

MD Simulations

All the components were described by the COMPASS force field. The BIN/PEI or LAM/BIN/PEI complex obtained from Adsorption module was employed to run molecular dynamic simulation. To eliminate unfavorable contacts, the initial configurations were subjected to 10000 steps of energy minimization with an energy convergence threshold of 10^{-4} Kcal/mol and a force convergence of 0.005 kcal mol^{-1} $Å^{-1}$. The van der Waals interactions were calculated with a cutoff of 12.5 Å, a spline width of 1 Å, and a buffer width of 0.5 Å, while the electrostatic interactions were estimated by the Ewald summation with an accuracy of 0.001 Kcal/mol. After minimization, the lowest energy configuration were chosen for simulation. 200 ns MD simulation using isothermal and isochoric (NVT) was carried out at 298 K using the Nose thermostat and 1 bar using the Andersen barostat for pure components.

DPD Simulation

Firstly, polymer blocks (PEI), BIN and water molecules were coarse-grained to corresponding beads (Figure S30). Flory−Huggins interaction parameters between these beads (Table S4) were calculated by Blends module in MS. Then a DPD simulation was activated at an ultra-fine level to investigate the self-assembly of BIN/PEI in a box of 100×100×100 rc^3 at the BIN/PEI/Water ratio of 1/1/8. According to the suggestion of Groot and Warren [1], the maximum repulsion between coarse grain i and j are obtained from Equation S1:

$$\alpha_{ij}(\rho=3)=78+3.25\chi_{ij} \qquad \text{(Equation S1)}$$

The repulsions α_{ij} at 298K used in this work were given in Table S5. Consequently, the size and number of the simulated aggregates were direct and important criteria to value the effects of simulation box size and integration time step. DPD simulations were performed in 100×100×100 r_c^3 with 20 ns. The DPD length unit or the cut-off radius r_c = 6.46 Å in our work. Thus, the box size in our work was characterized by effective dimensions of 1938Å×1938Å×1938 Å. All the simulations were performed using Mesocite program incorporated in Materials Studio 2017 at 298 K, and total simulation time is 20 ns with integration time step of 150 fs.

Preparation of Cy5.5 labeled nanoparticles

To synthesize Cy5.5-conjugated PEI (Cy5.5-PEI), 15 mg Cy5.5 NHS ester in 5 mL of DMSO was added into 5 mL of DMSO containing 160 mg PEI, the obtained solution was stirred at 40 °C for 12 h in dark. After complete reaction, Cy5.5-PEI were obtained by dialysis of the solution against deionized water for 24 h. Then 5 mg Cy5.5-PEI and 5 mg BIN were firstly dissolved in 1 mL DMSO to dialyze against deionized water at 25 °C. The deionized water was exchanged every 2 h. After 12 h of dialysis, Cy5.5-pBIN were obtained by collecting the solution in the dialysis bag without further treatment. For preparing Cy5.5 labeled LApBIN, 0.1 mL LAM solution (10 mg/mL) was added into 1 mL Cy5.5-pBIN suspension (1 mg/mL), and then incubated for 1 min under ultrasonic vibration. The unabsorbed LAM was removed by twice water washing after high speed centrifugation at 12000 rpm.

Characterization of various nanoparticles

Dynamic light scattering (DLS) and ξ-potential measurements were carried out on a Malvern Zetasizer Nano ZS instrument at room temperature. Transmission electron microscopy (TEM) scan to observe the morphology of nanoparticles was performed on a Tecnai-10 microscope (Philips, the Netherlands) operating at an acceleration voltage of 80 kV.

In vitro release study

1 mg pBIN NPs or LApBIN NPs were dispersed into 1 mL of deionized water, and then put into a dialysis membrane bag (MWCO 1000 Da). The dialysis membrane bags were immersed into vials containing 25 mL of PBS in a shaking bed at 37 °C. Tween 80 (0.1 % v/v) was added into the release medium to improve the solubility of BIN in PBS solution (pH 7.4, 0.01 M). The amount of released drug was detected by HPLC. The detailed HPLC method for determining BIN was provided in Supplementary Materials.

Cell culture

Raw 264.7 mouse macrophage was obtained from the Cell Bank of the Committee on Type Culture Collection of Chinese Academy of Sciences (Shanghai, China). The cells were cultured in RPMI 1640 medium containing 10 % FBS, 100 U/mL penicillin, and 100 μg/mL streptomycin. All cells were incubated at 37 °C in a humidified atmosphere with 5 % CO_2 for 24 h before further experiments.

Migration of macrophages after LApBIN internalization

Raw 264.7 cells were seeded into 6-well plates at 3×10^5 cells per well in 2 ml of growth medium. LApBIN were added in each well at 2.5 μg/mL, and incubated for 4 h. The cells were washed with PBS, detached by trypsin digestion, centrifuged, and re-suspended in the medium containing 0.5% FBS. Then, these Raw 264.7 cells were seeded into the upper chambers of Transwell (6.5 mm Transwell® with 5.0 μm pore polycarbonate membrane, Corning) and cultured with 100 μL of medium. In the lower chambers, 600 μL of medium supplemented with 3×10^5 cells of foam cells or endothelial cells was added. After incubation for 6 h at 37 °C, the cells were fixed, and stained with crystal violet. The upside membrane surface was scraped to remove

remaining cells, and the cells migrated to the downside surface were counted in four high-power fields per membrane under a microscope.

Supplementary tables

Table S1. The adsorption energies of BIN on PEI predicated by Adsorption Module. (Unit: Kcal/mol)

**Structures**	**Total energy**	**Adsorption energy**	**Rigid adsorption energy**	**Deformation energy**	**Bindarit : dEad/dNi**
PEI-BIN- 1	29.64	-317.74	-30.74	-287.01	-317.74
PEI-BIN- 2	30.37	-317.02	-29.25	-287.77	-317.02
PEI-BIN- 3	33.94	-313.44	-26.87	-286.58	-313.44
PEI-BIN- 4	34.18	-313.21	-26.69	-286.52	-313.21
PEI-BIN- 5	36.19	-311.20	-23.44	-287.75	-311.20
PEI-BIN- 6	36.73	-310.66	-23.18	-287.48	-310.66
PEI-BIN- 7	37.00	-310.39	-23.73	-286.66	-310.39
PEI-BIN- 8	37.29	-310.10	-24.42	-285.67	-310.10
PEI-BIN- 9	37.50	-309.89	-24.16	-285.73	-309.89
PEI-BIN- 10	37.70	-309.69	-21.87	-287.82	-309.69
Average	35.05	-312.33	-25.43	-286.90	-312.33

Table S2. The adsorption energies of PEI fragment (Degree of Polymerization =8) on PEI predicated by Adsorption Module.

(Unit: Kcal/mol)

**Structures**	**Total energy**	**Adsorption energy**	**Rigid adsorption energy**	**Deformation energy**	**PEI fragment : dEad/dNi**
PEI-PEIF- 1	-8.21	-207.88	-32.28	-175.60	-207.88
PEI-PEIF- 2	-2.33	-202.00	-25.71	-176.29	-202.00
PEI-PEIF- 3	-2.06	-201.72	-25.28	-176.45	-201.72
PEI-PEIF- 4	-1.22	-200.89	-25.40	-175.49	-200.89
PEI-PEIF- 5	-0.78	-200.45	-25.88	-174.57	-200.45
PEI-PEIF- 6	-0.40	-200.07	-27.18	-172.89	-200.07
PEI-PEIF- 7	0.48	-199.19	-26.30	-172.89	-199.19
PEI-PEIF- 8	2.09	-197.58	-20.18	-177.40	-197.58
PEI-PEIF- 9	2.29	-197.38	-20.24	-177.14	-197.38
PEI-PEIF- 10	3.14	-196.52	-19.64	-176.88	-196.52
Average	-0.70	-200.37	-24.81	-175.56	-200.37

Table S3. The adsorption energies of LAM on BIN/PEI complex predicated by Adsorption Module.

(Unit: Kcal/mol)

Structures	Total energy	Adsorption energy	Rigid adsorption energy	Deformation energy	PEI fragment : dEad/dNi
BIN/PEI- LAM - 1	196.28	-915.24	-61.42	-853.82	-915.24
BIN/PEI- LAM - 2	196.85	-914.68	-60.21	-854.47	-914.68
BIN/PEI- LAM - 3	197.10	-914.43	-60.27	-854.15	-914.43
BIN/PEI- LAM - 4	197.56	-913.96	-60.34	-853.62	-913.96
BIN/PEI- LAM - 5	198.36	-913.16	-58.21	-854.95	-913.16
BIN/PEI- LAM - 6	198.81	-912.71	-59.92	-852.80	-912.71
BIN/PEI- LAM - 7	199.17	-912.35	-59.93	-852.42	-912.35
BIN/PEI- LAM - 8	200.05	-911.47	-59.96	-851.52	-911.47
BIN/PEI- LAM - 9	201.26	-910.27	-57.43	-852.84	-910.27
BIN/PEI- LAM -10	202.14	-909.38	-58.03	-851.36	-909.38
Average	198.76	-912.77	-59.57	-853.19	-912.77

Table S4. The Flory−Huggins interaction parameters calculated by Blends

(Unit: Kcal/mol)

Base	Screen	Chi (298 K)	E_{mix} (298 K)	E_{bb} avg (298 K)	E_{bs} avg (298 K)	E_{ss} avg (298 K)
Bindarit	PEI	-20.4712	-12.1228	-12.8628	-11.3123	-5.42661
Bindarit	water	26.60149	15.75307	-12.8628	-3.74275	-2.33172
EI	water	1.599389	0.947138	-2.44349	-1.97868	-2.33135
LAMs	PEI	-4.14983	-2.45748	-9.98446	-6.86706	-2.9227
LAMs	water	-13.358	-7.91041	-9.98446	-6.35734	-2.33434
LAMs	Bindarit	-6.49692	-3.84739	-9.98446	-11.2457	-11.4781

Table S5. The interaction parameters used in DPD simulations (unit: kT)..

α_{ij}	BIN	PEI	LAM	Water
**BIN**	78			
**PEI**	11.47	78		
**LAM**	56.89	64.51	78	
**Water**	164.46	83.20	34.59	78

Supplementary figures

A B C

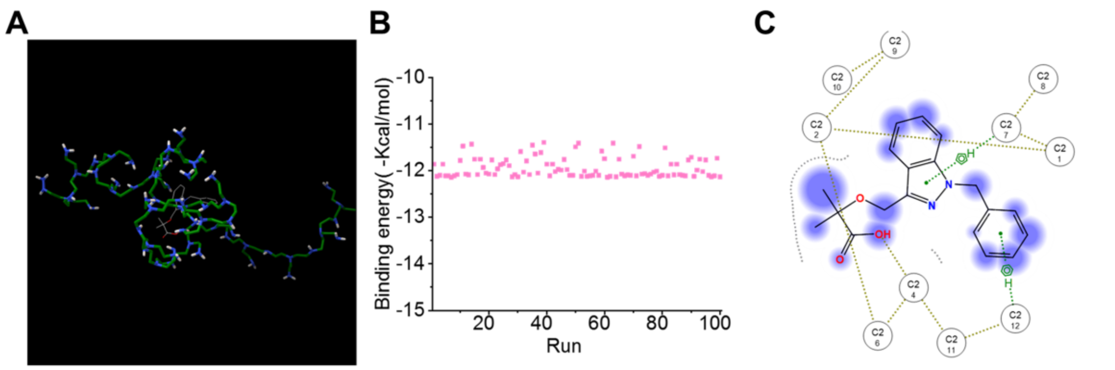

Figure S1. The dock results of BIN to PEI. (A)The lowest 3D conformation obtained from Autodock. (B) The distribution of binding energy during the dock runs. (C) The 2D diagram of the interaction between BIN and PEI.

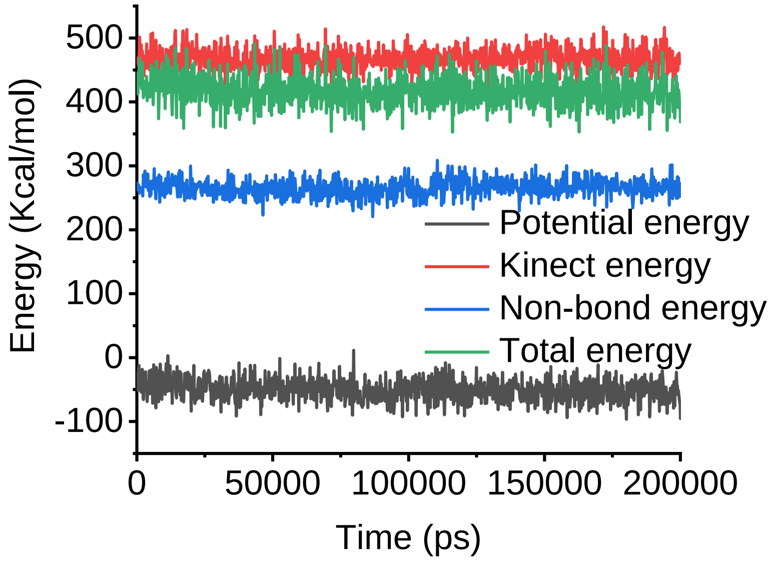

Figure S2. The energy changes during the MD simulation of BIN/PEI complex.

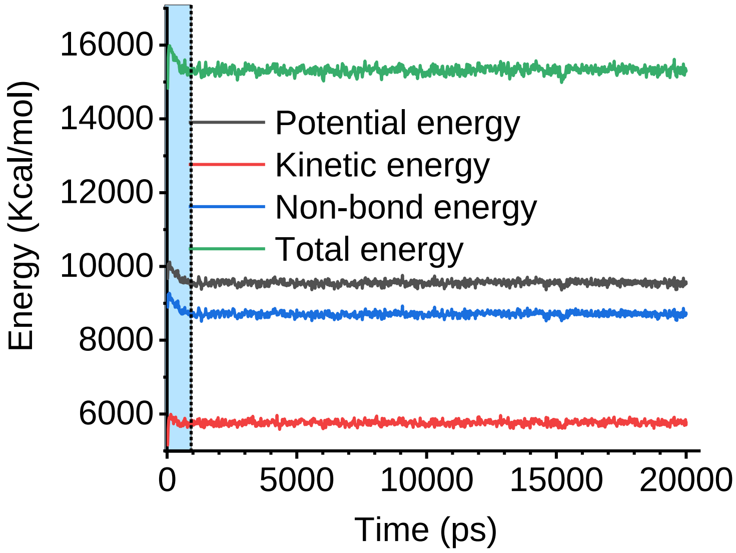

Figure S3. The energy changes during the 20 ns of BIN/PEI DPD simulation.

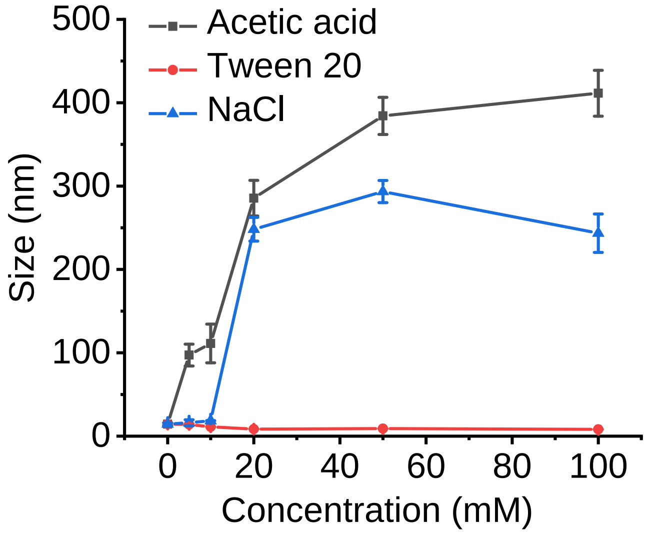

Figure S4. Dissociation behaviors of LApBIN after dispersion in Acetic acid, Tween 20 and NaCl.

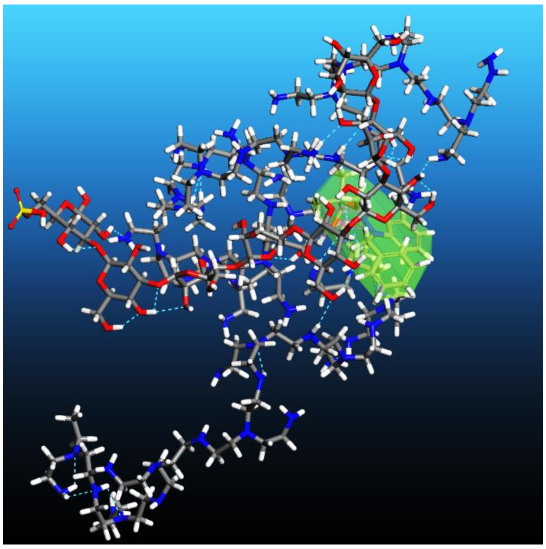

Figure S5. The 3D conformation of the LAM/BIN/PEI complex obtained from Adsorption module.

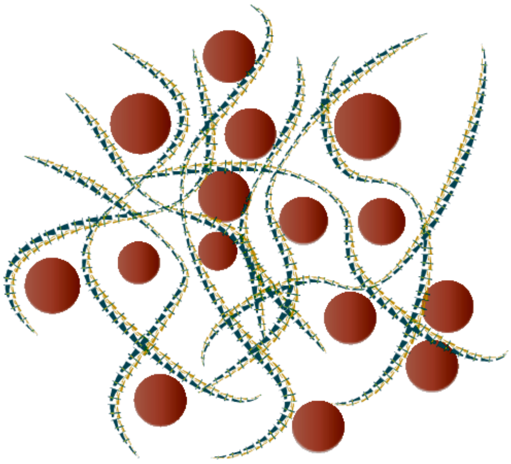

Figure S6. Schematic diagram of LApBIN formation.

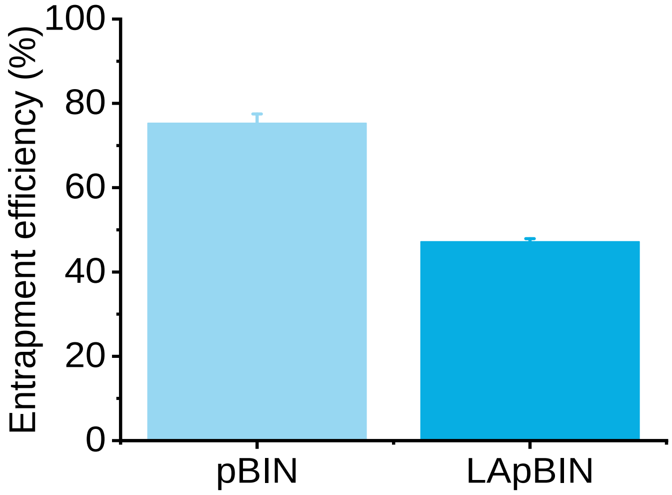

Figure S7. The drug entrapment efficiency of pBIN and LApBIN.

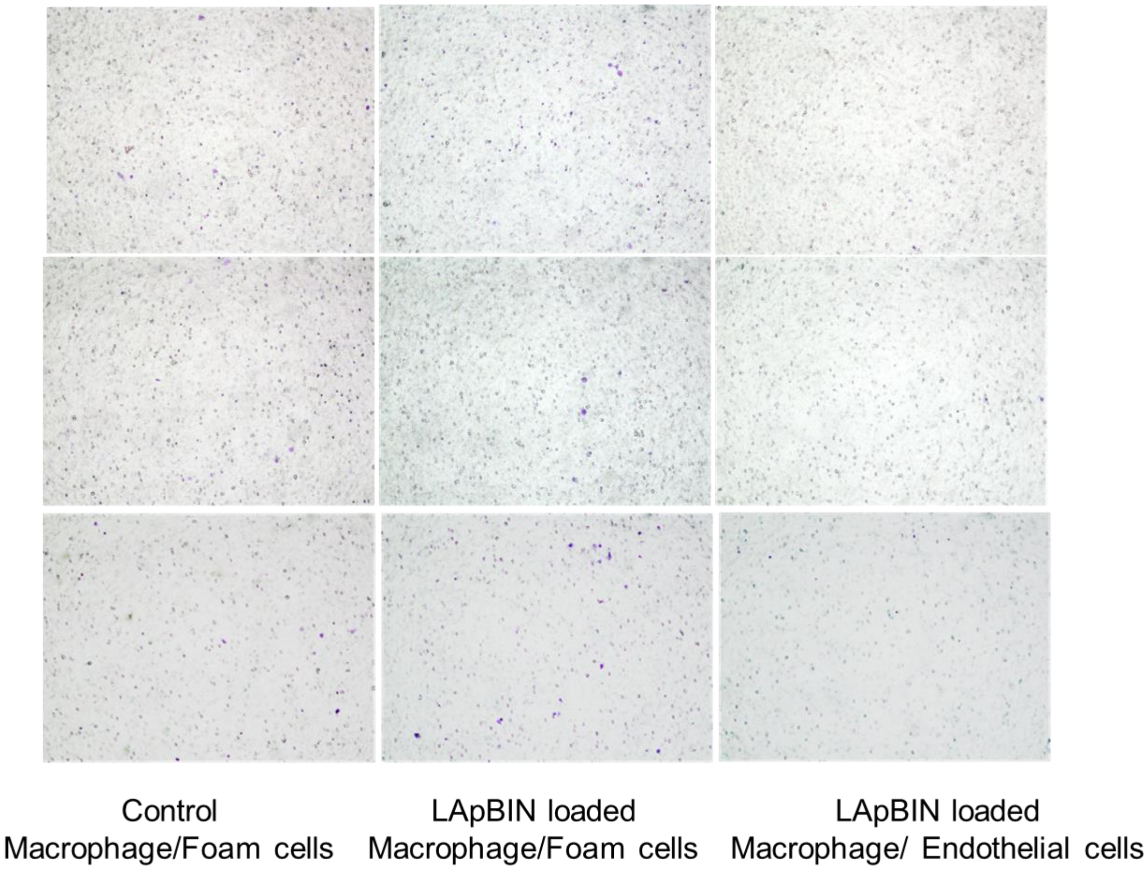

Figure S8. The migration of Raw 264.7 cells in various co-incubation systems.

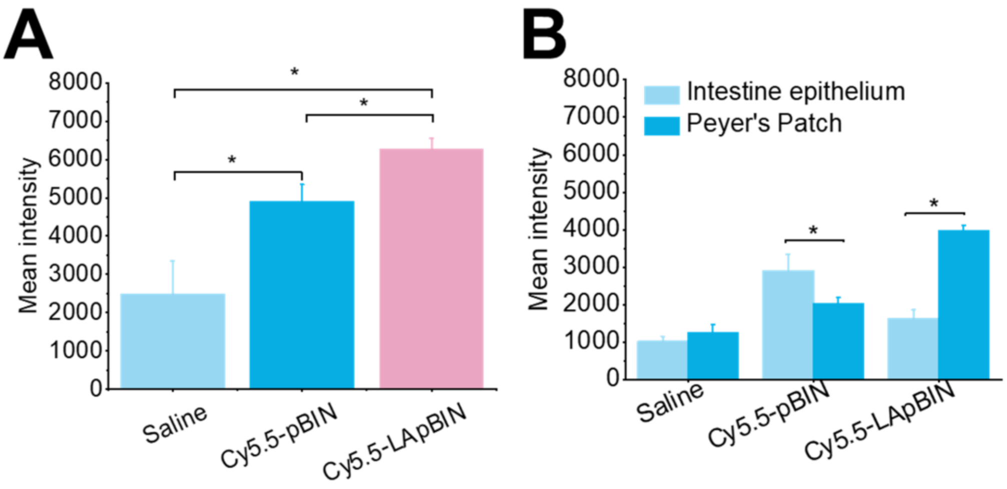

Figure S9. The quantitative analysis of *ex vivo* images of gut tissues (A) and isolated intestine epithelium and Peyer's Patch (B) after oral administration of Cy5.5 labeled nanoparticles in DIO mice. * means $p < 0.05$.

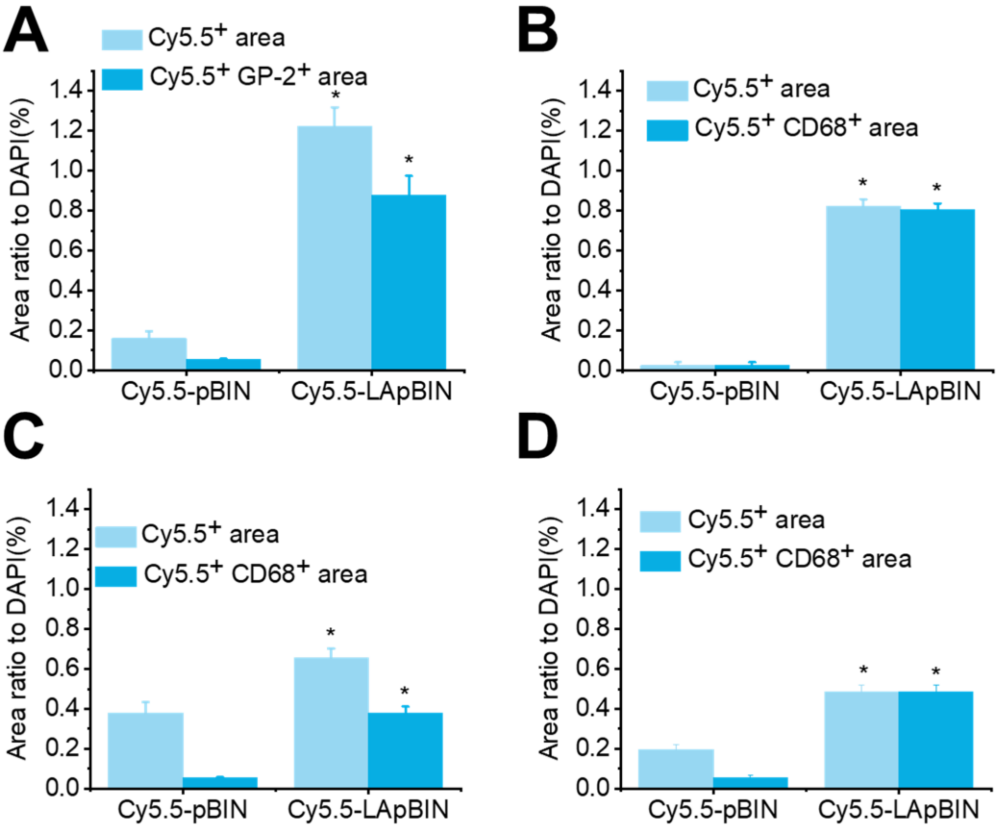

Figure S10. The quantitative analysis of Cy5.5 distribution in Peyer's Patch (A), adipose tissues (B), fatty liver (C) and aorta tissues (D), after oral administration of Cy5.5 labeled nanoparticles in DIO mice. * means $p < 0.05$.

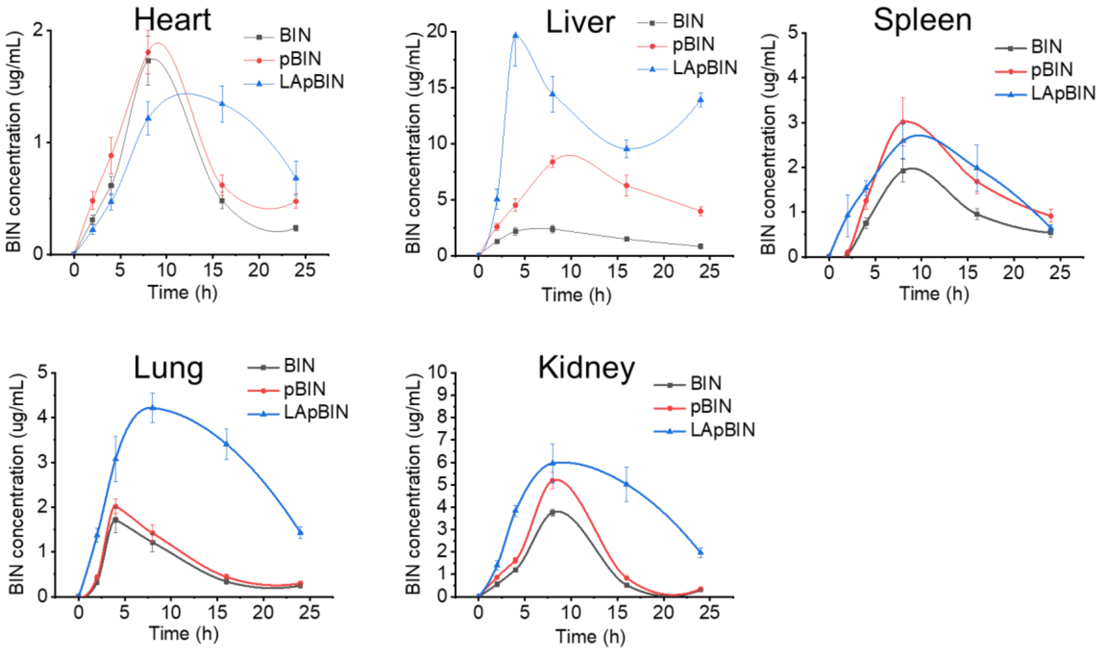

Figure S11. The dynamic concentrations of BIN from different BIN formulations in major organs.

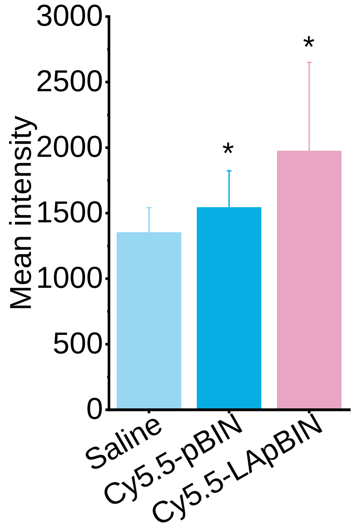

Figure S12. The quantitative analysis of *ex vivo* images of adipose tissues after oral administration of Cy5.5 labeled nanoparticles in DIO mice. * means vs saline group, p <0.05.

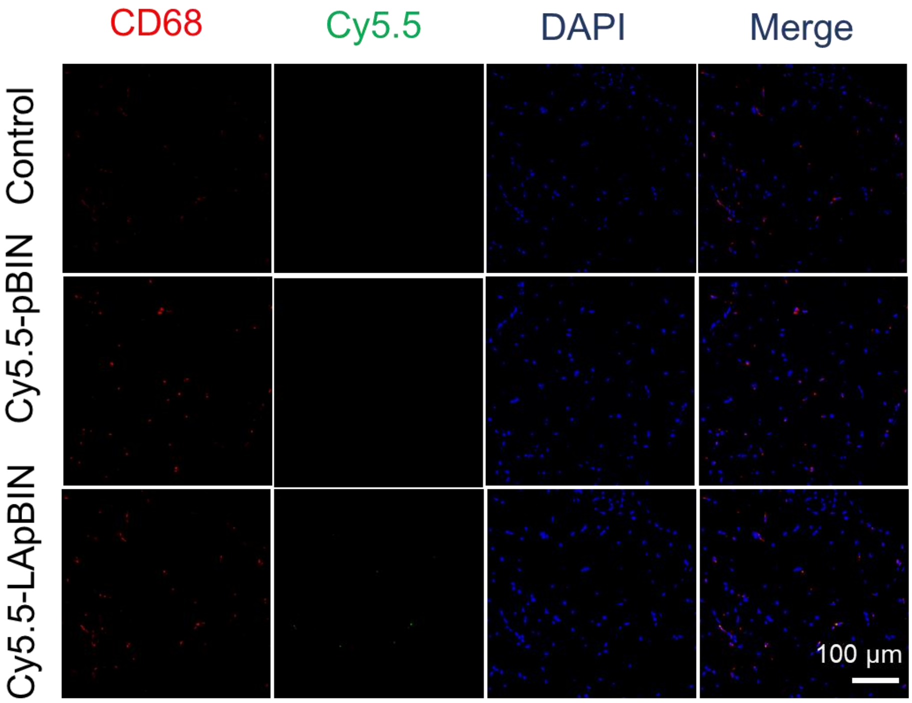

Figure S13. Co-localization of Cy5.5 (green) and macrophage marker CD68 (red) in adipose tissues.

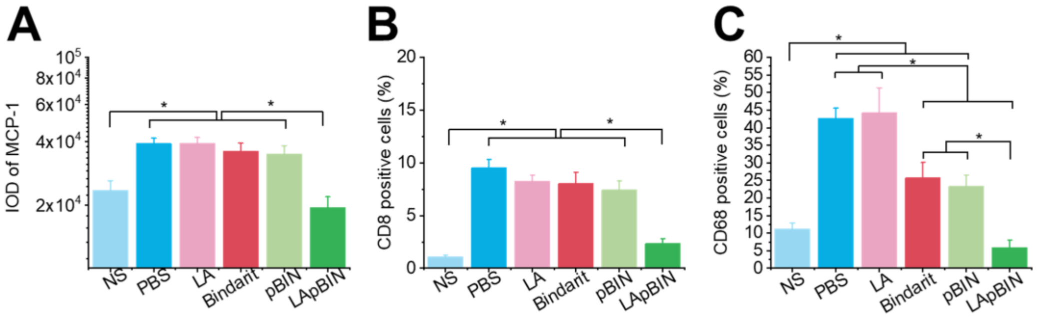

Figure S14. The quantitative analysis of immunofluorescence or immunohistochemistry section of adipose tissue. * means $p < 0.05$.

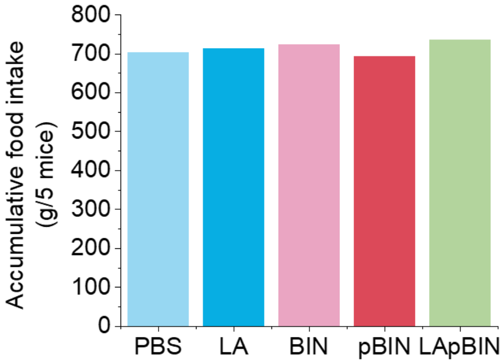

Figure S15. The accumulative food intake of mice in different groups.

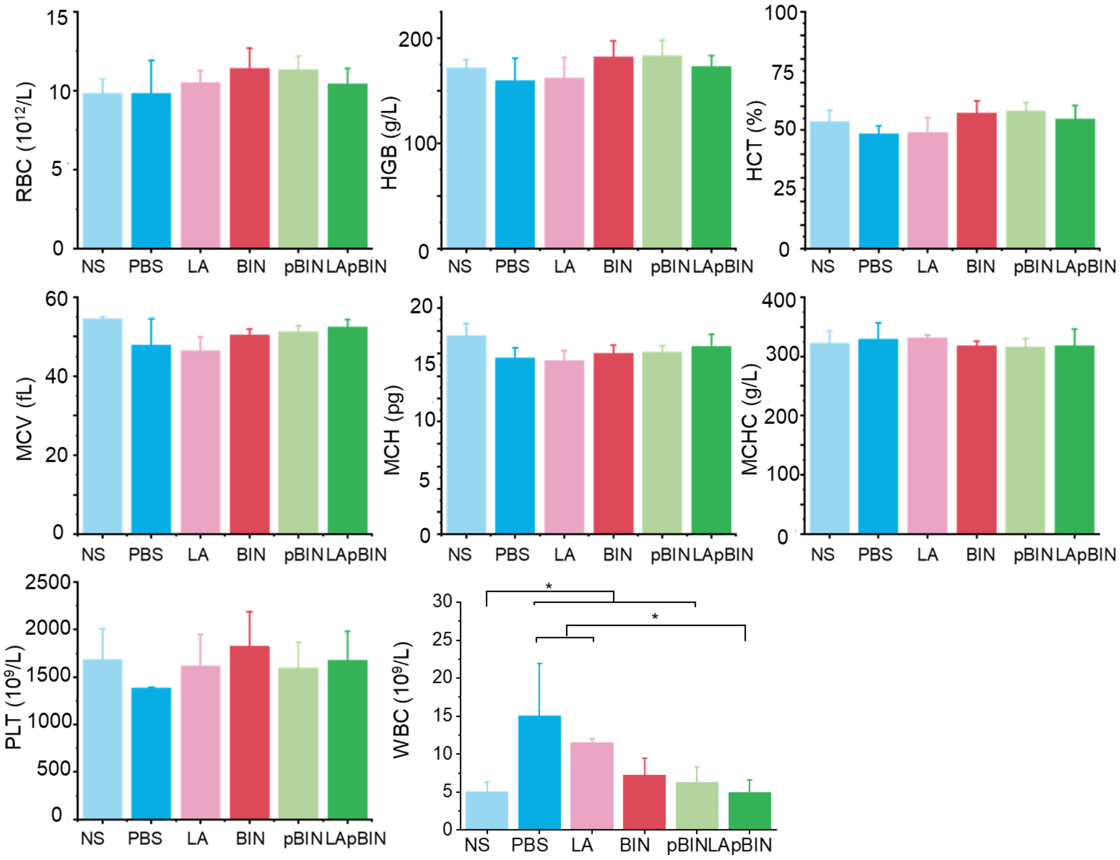

Figure S16. Main hematological parameters of blood samples, including red blood cells (RBC), hemoglobin (HGB), hematocrit (HCT), mean red blood cell volume (MCV), mean corpuscular hemoglobin (MCH), platelets (PLT), and white blood cells (WBC) from DIO mice subjected to various treatments. * means $p<0.05$.

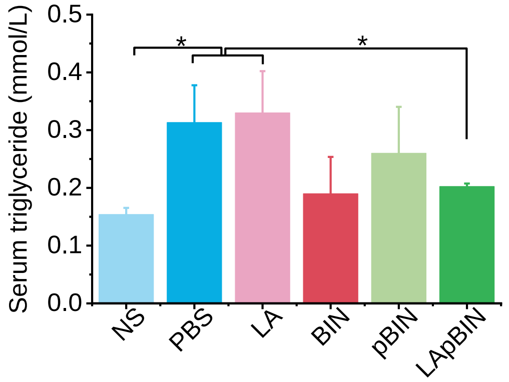

Figure S17. The concentrations of serum triglyceride of DIO mice subjected to various treatments. * means p<0.05.

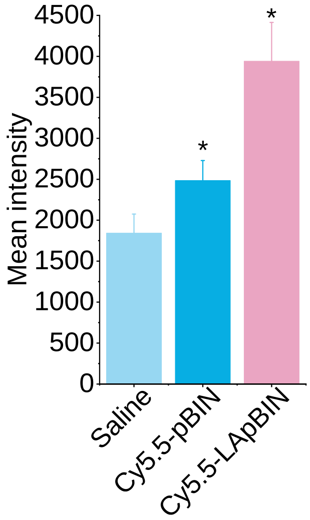

Figure S18. The quantitative analysis of *ex vivo* images of fatty liver tissues after oral administration of Cy5.5 labeled nanoparticles in DIO mice. * means vs saline, $p < 0.05$.

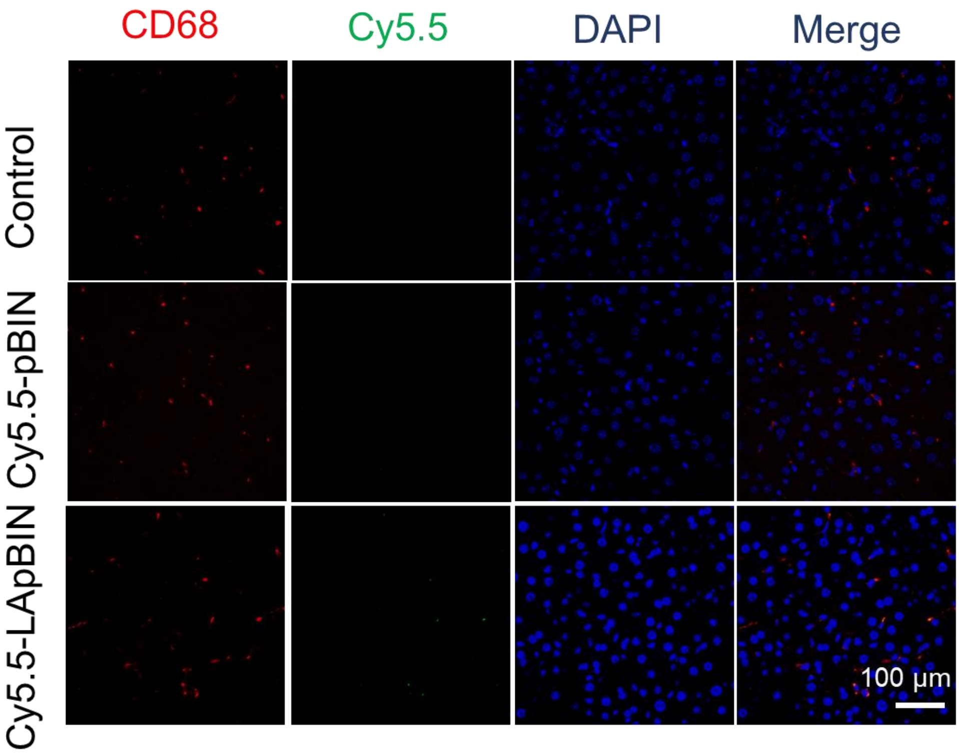

Figure S19. Co-localization of Cy5.5 (green) and macrophage marker CD68 (red) in fatty liver.

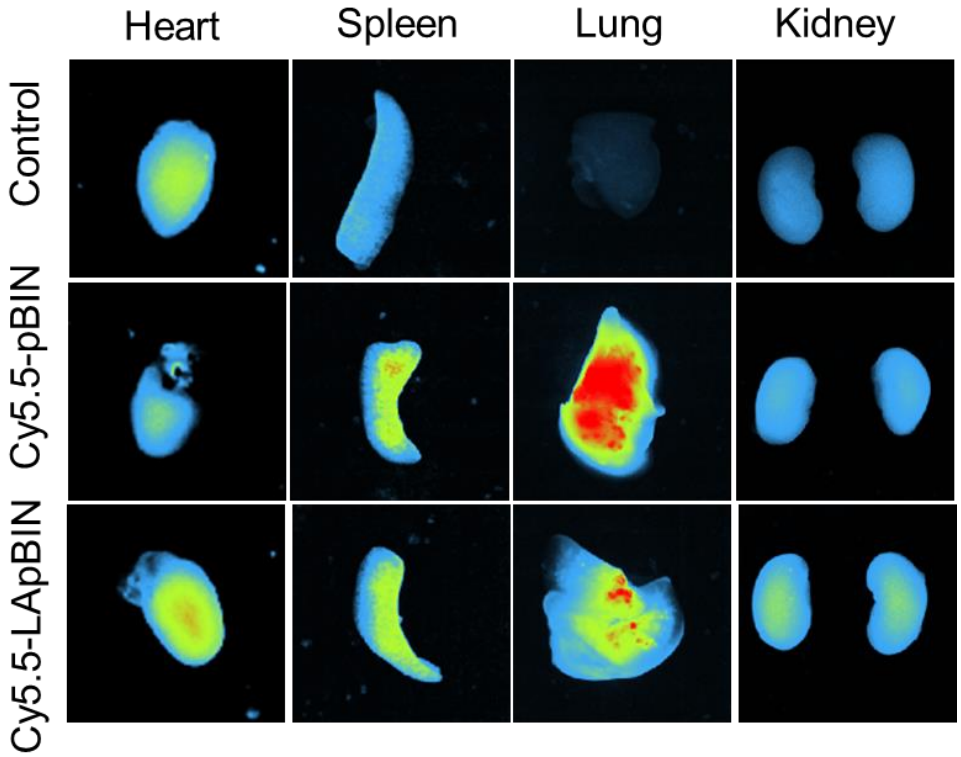

Figure S20. *Ex vivo* images of Cy5.5 distribution in heart, spleen, lung and kidney.

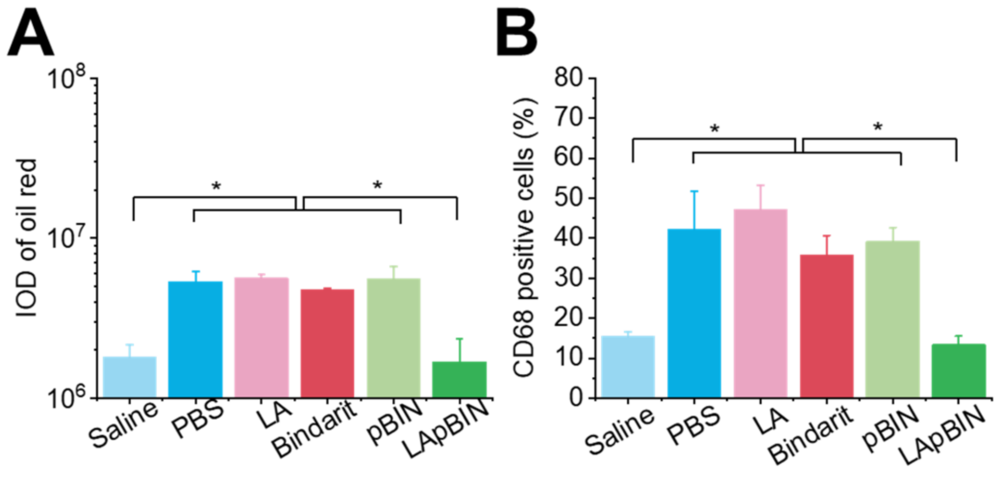

Figure S21.The quantitative analysis of oil red and CD68 in fatty liver. * means $p < 0.05$.

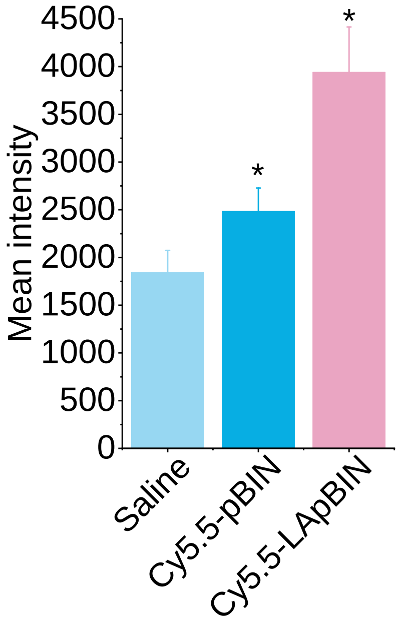

Figure S22. The quantitative analysis of *ex vivo* images of aorta tissues after oral administration of Cy5.5 labeled nanoparticles in $ApoE^{-}$ mice. * means vs saline, $p < 0.05$.

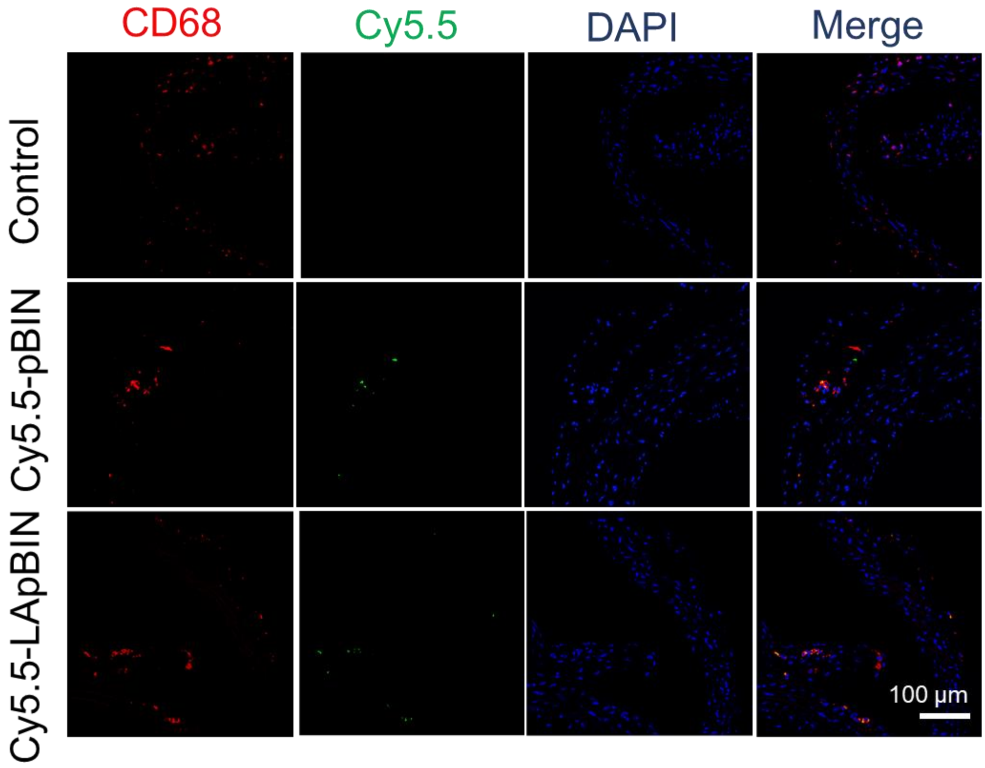

Figure S23. Co-localization of Cy5.5 (green) and macrophage marker CD68 (red) in aorta tissues.

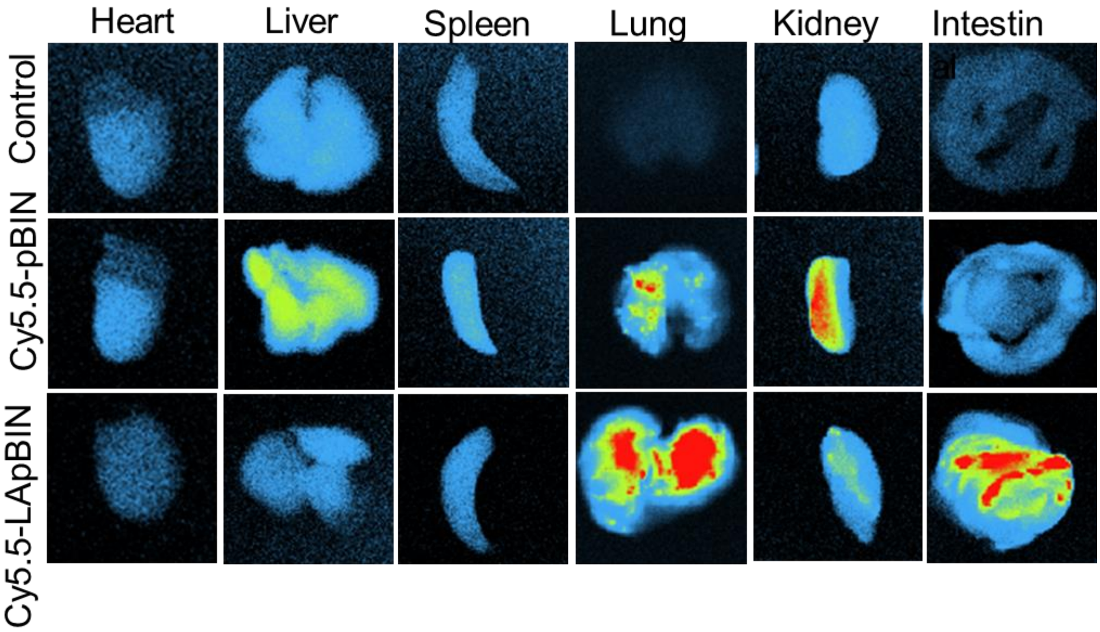

Figure S24. *Ex vivo* images of Cy5.5 distribution in heart, liver, spleen, lung, kidney and intestinal from ApoE^{-} mice.

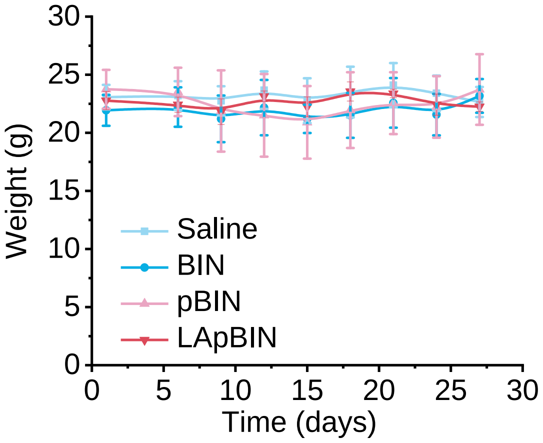

Figure S25. The weight growth of mice during receiving treatments.

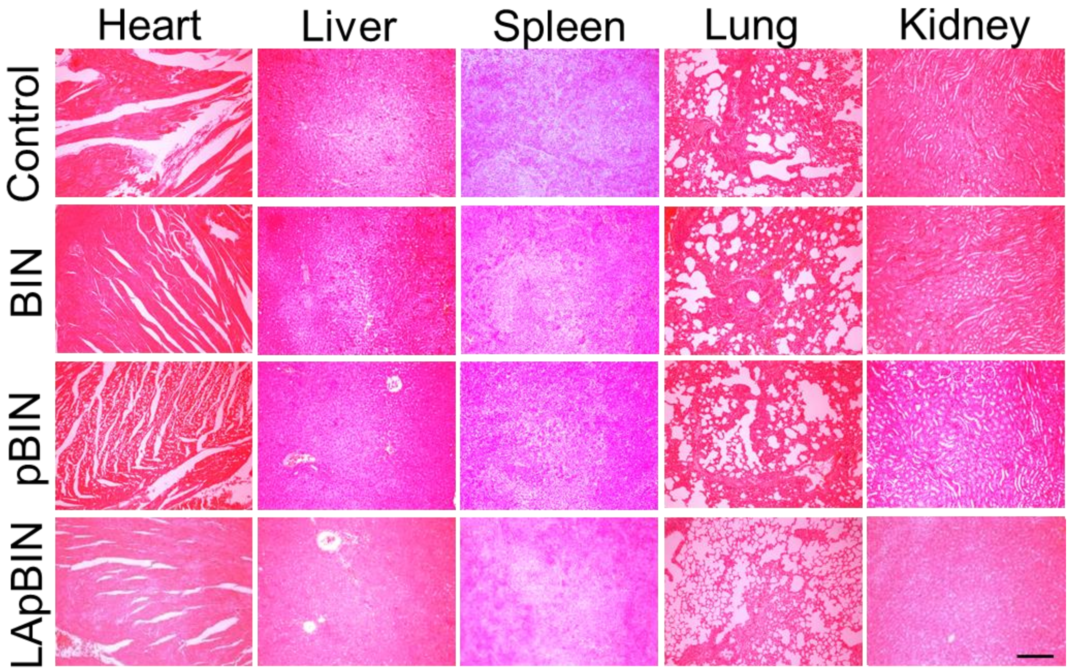

Figure S26. The histopathological sections of major organs resected from ApoE^{-} mice after oral administration of various BIN formulations for 30 days. Scale bar means 200 μm.

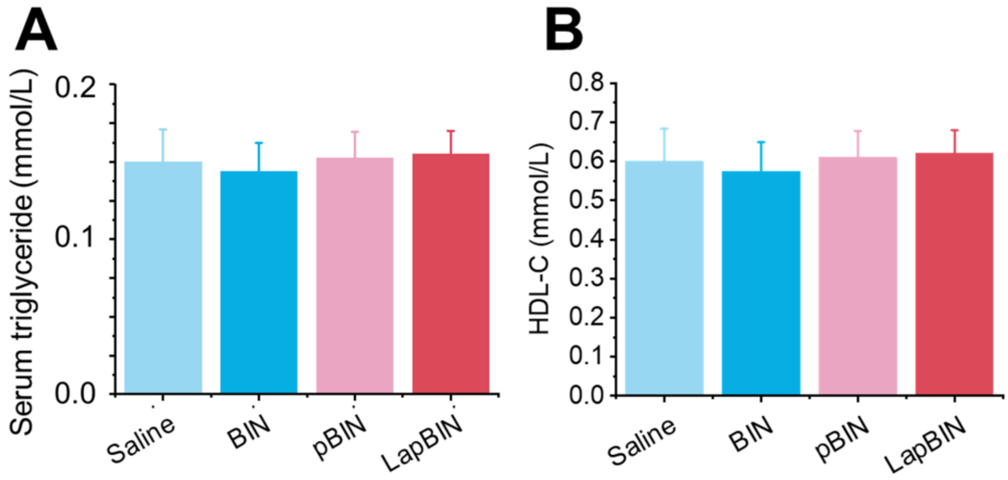

Figure S27. The concentration of triglycerides and high-density lipoprotein (HDL-C) in blood collected from ApoE^{-} mice after oral administration of various BIN formulations for 30 days.

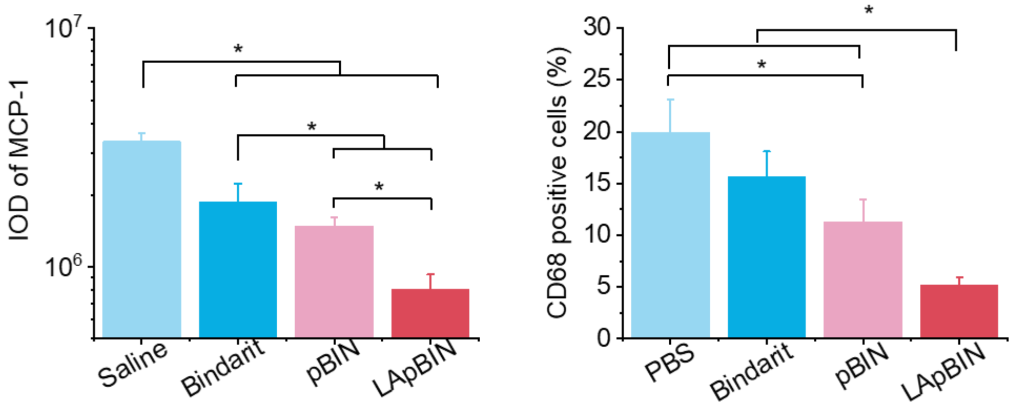

Figure S28. The quantitative analysis of MCP-1 and CD68⁺ cells of aorta tissues. * means p <0.05.

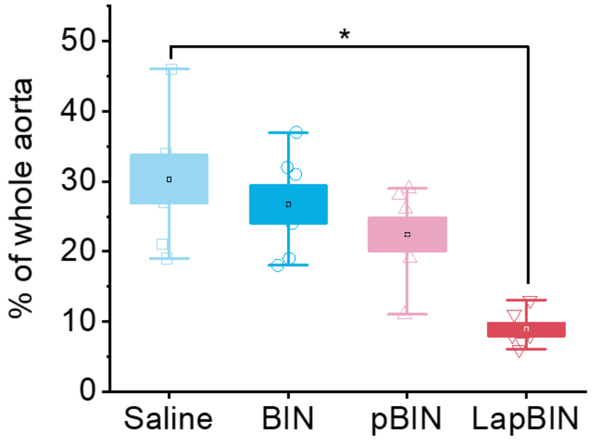

Figure S29. The calculated plaque percent of whole aorta resected from ApoE^{-} mice after oral administration of various BIN formulations for 30 days. * means $p < 0.05$.

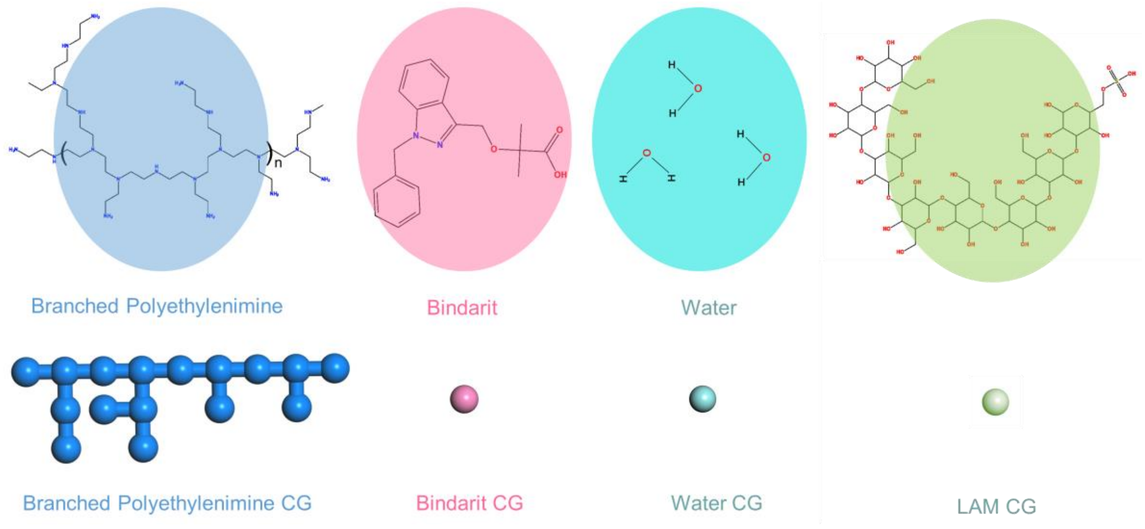

Figure S30. Coarse-grained models of PEI, BIN, LAM and H_2O.

Supplementary references

1. Groot, R.D. and P.B. Warren, Dissipative particle dynamics: Bridging the gap between atomistic and mesoscopic simulation. J CHEM PHYS. 1997; 107(11): 4423-4435.
